# EMAF-Net: A Lightweight Single-Stage Detector for 13-Class Object Detection in Agricultural Rural Road Scenes

**DOI:** 10.3390/s26072055

**Published:** 2026-03-25

**Authors:** Zhixin Yao, Chunjiang Zhao, Yunjie Zhao, Xiaoyi Liu, Tuo Sun, Taihong Zhang

**Affiliations:** 1 College of Computer and Information Engineering, Xinjiang Agricultural University, Urumqi 830052, China; 320192868@xjau.edu.cn (Z.Y.); 320200031@xjau.edu.cn (Y.Z.); 320243423@stu.xjau.edu.cn (X.L.); 320243412@stu.xjau.edu.cn (T.S.); 2 Xinjiang Agricultural Informatization Engineering, Technology Research Center, Urumqi 830052, China; 3 Research Center for Intelligent Agriculture, Ministry of Education Engineering, Urumqi 830052, China; 4 National Engineering Research Center for Information Technology in Agriculture, Beijing 100125, China

**Keywords:** rural road, agricultural machinery automatic navigation, object detection, EMAF-Net, multi-head self-attention mechanism, multi-scale feature fusion

## Abstract

**Highlights:**

**What are the main findings?**

- Developed a lightweight object detection model integrating EfficientNet-B1 with multi-head self-attention (EMHA) for enhanced global context modeling in rural road scenarios.
- Proposed an Improved ASPP module combining atrous convolution and SPP-like pooling with bidirectional FPN for robust multi-scale feature fusion and small-object detection.
- Achieved superior detection performance with mAP@0.5 of 64.05% and mAP@0.5:0.95 of 48.95%, while maintaining low computational cost (38.5 GFLOPs) and high real-time capability (184.62 FPS).

**What are the implications of the main findings?**

- Enables accurate and real-time detection of multiple object categories (person, animal, agricultural machinery, traffic light, etc.) in complex rural environments, supporting safe autonomous navigation and obstacle avoidance for agricultural vehicles.
- Provides a computationally efficient solution suitable for edge deployment on resource-constrained platforms, facilitating practical application in precision agriculture and smart farming systems.

**Abstract:**

Rural road perception for agricultural machinery automation faces challenges including complex backgrounds, drastic lighting and weather variations, frequent occlusions, and high densities of small objects with significant scale variations. These factors make conventional detectors prone to missed detections and misclassifications. To address these issues, a 4K rural road dataset with 4771 images is constructed. The dataset covers 13 object categories and includes diverse day/night conditions and multiple weather scenarios on both structured and unstructured roads. EMAF-Net, a lightweight single-stage detector based on YOLOv4-P6, is proposed. The backbone integrates an EMHA module combining EfficientNet-B1 with multi-head self-attention (MHSA) for enhanced global context modeling while preserving efficient local feature extraction. The neck adopts an Improved ASPP and a bidirectional FPN to achieve robust multi-scale feature fusion and expanded receptive fields. Meanwhile, CIoU loss is used to optimize bounding box regression accuracy. The experimental results demonstrate that EMAF-Net achieves an mAP@0.5 of 64.05% and an mAP@0.5:0.95 of 48.95% on a rural road dataset. At the same time, it maintains a lightweight design with 18.3 M parameters and a computational complexity of 38.5 GFLOPs. Ablation studies confirm the EMHA module contributes a 6.22% mAP@0.5 improvement, validating EMAF-Net’s effectiveness for real-time rural road perception in autonomous agricultural systems.

## 1. Introduction

In agricultural production, automatic navigation technology for agricultural machinery is gradually becoming the key to improving operational efficiency and accuracy, with reliable environmental perception capability as the core prerequisite [1]. With the continuous development of modern autonomous driving technology, object detection technology based on computer vision and deep learning has been widely applied in agricultural automation, especially in complex rural road scenarios [2]. The automatic navigation of agricultural machinery not only enhances operational efficiency and reduces labor costs, but also reduces safety risks caused by human operational errors [3].

However, compared with urban roads, rural roads often exhibit stronger unstructured characteristics and higher uncertainty. Road materials are diverse, including asphalt roads, non-hardening paths, and cement roads. Meanwhile, the background often contains complex textures such as farmland vegetation, soil dust, and scattered debris, which introduce strong visual interference [4]. Lighting and weather conditions also vary significantly, including strong sunlight, shadows, rain, fog, and weak illumination at night. Additionally, rural roads often feature mixed traffic involving humans, vehicles, and animals, as well as unique objects such as agricultural machinery and tools, with frequent occlusions and significant scale variations. These factors impose higher demands on multi-object detection and dynamic obstacle perception in the autonomous driving scenarios of agricultural machinery, especially the need to balance high precision and real-time performance under the limited computational resources of edge devices [5]. Our previous work focused on semantic segmentation of rural road scenes [6]. Semantic segmentation provides pixel-level scene understanding, enabling the delineation of drivable areas, background vegetation, and static infrastructure within rural road environments. This level of granularity facilitates comprehensive environmental perception but is inherently limited in its ability to distinctly identify and localize individual dynamic obstacles such as humans, animals, and agricultural equipment, which frequently appear in rural road scenarios. Object detection addresses this limitation by focusing on the rapid and precise localization of discrete objects, generating bounding boxes and class labels for real-time identification and tracking of potential hazards. In the context of autonomous agricultural machinery, combining pixel-level context from segmentation with the instance-level discrimination of object detection supports both holistic scene understanding and the immediate recognition of critical dynamic obstacles. This integration is essential for safe navigation, collision avoidance, and efficient task execution under complex, unstructured rural conditions.

In recent years, single-stage object detection methods (such as the YOLO series) have been widely applied in real-time scenarios due to their fast end-to-end training and inference speeds. However, their convolutional backbone networks predominantly rely on local operators, which limits their ability to effectively model long-range dependencies. Consequently, such methods are prone to missed detections and category confusion when addressing distant small objects, densely packed objects, and scenes with complex texture interference. On the other hand, Transformer-based detection methods can capture global semantic relationships through attention mechanisms. However, they typically involve high computational costs. As a result, they are difficult to directly deploy on resource-constrained agricultural machinery platforms. Therefore, an essential challenge for rural road detection is how to integrate the efficient local representation of convolution with the global modeling capability of attention mechanisms under lightweight constraints. In addition, the quality of multi-scale feature alignment and fusion must also be improved.

Furthermore, publicly available detection data and evaluation benchmarks for rural roads remain relatively scarce. Existing representative datasets primarily focus on urban structured roads and provide limited coverage of nighttime and adverse weather conditions. In addition, they lack key categories relevant to agricultural scenarios, such as agricultural machinery, farm tools, and free-range animals. This absence of representative training and testing data not only limits the generalization ability of models in agricultural scenarios but also hinders the establishment of a unified standard for algorithm reliability assessment in real-world applications.

Therefore, in view of the problems of complex backgrounds, diverse illumination, road dirt cover, narrow roads, many bends and many types of irregular obstacles in the rural road environment, this paper first constructs a rural road object detection dataset covering 13 types of objects and containing 4K images. A lightweight and efficient detection network, EMAF-Net, for agricultural machinery automatic driving was proposed. Based on YOLOv4-P6 [7], this method introduces an EMHA module composed of EfficientNet-B1 and multi-head self-attention into the backbone network to improve the discrimination and global correlation of feature expression. In the neck, the Improved ASPP and FPN are used for multi-scale fusion to enhance the robustness of the receptive field and scale, and the CIoU loss is used to optimize the localization accuracy. Experiments were carried out on the self-built dataset and BDD100K [8]. The results show that the proposed method achieves a good balance between accuracy, computational complexity and real-time performance. The proposed approach provides a reliable perception solution for autonomous agricultural machinery operating in complex rural road environments, which may contribute to improving operational safety, reducing labor dependence, and promoting the development of intelligent agricultural transportation systems. This is particularly important for large-scale agricultural production and remote rural areas facing labor shortages. The main contributions of this paper can be summarized as follows.

1. An object detection dataset for rural road scenarios in Xinjiang, China was constructed, covering multiple time periods, various weather conditions and different road surface types. It includes 4K resolution images and 13 types of agricultural-related objects. A data partitioning strategy based on region hash bucketing and a strict IoU consistency (IoU ≥ 0.75) annotation quality control process were proposed.
2. Proposing a lightweight detection network with a hybrid architecture, EMAF-Net: a network that replaces the traditional convolutional backbone with the EMHA module, while maintaining the P6 structure and real-time performance to enhance the global semantic modeling ability, and adopts Improved ASPP and FPN to improve the multi-scale detection performance.
3. Comparative experiments and ablation experiments were conducted on the self-built dataset and BDD100K to demonstrate the overall advantages of the proposed method in terms of accuracy, computational complexity, and inference speed. Moreover, the influence of annotation strictness and feature layer selection strategies on detection performance was analyzed.

## 2. Related Work

In the field of computer vision within artificial intelligence, there are many methods that can handle images and videos of various clarity levels and some that are already distorted. For instance, intelligent video surveillance systems require effective techniques to accurately and quickly detect objects. They utilize convolutional neural networks to process images of different sizes and clarity levels. The object detection technology based on convolutional neural networks is typically divided into two categories: one is the two-stage object detection model based on candidate regions, and the other is the one-stage detection model that can be performed in real-time. The former divides the object detection task into two stages. First, it selects a group of potential regions containing the object through the candidate region network and then further classifies these regions to obtain the final object detection result. Representative models include Faster R-CNN, R-FCN, Mask R-CNN, and RepPoints, etc. The latter directly uses the global features extracted by the convolutional neural network (CNN) for the positioning and classification of the object. This type of model has a higher inference rate than the two-stage detection algorithm, but the accuracy is slightly lower. Representative models include YOLO, SSD, RetinaNet, and CornerNet, etc. [9].

Among these single-stage detectors, the YOLO series has attracted significant attention due to its favorable balance between detection accuracy and inference speed, making it widely adopted in real-time perception tasks. This model was first proposed by Redmon et al. [10] in 2016 as the single-stage object detection model YOLOv1, laying the foundation for the subsequent YOLO series. However, the efficiency of its backbone and neck is still relatively low, and the model lacks modules with attention mechanisms. Such modules have been widely applied in computer vision tasks based on convolutional neural networks, integrating contextual information to enable the model to extract more useful feature information and discard redundant feature information. The absence of these contents has limited the fitting ability and inference speed of the network to a certain extent. To further improve detection accuracy and robustness, a number of studies have proposed alternative single-stage detection architectures or optimization strategies. In 2017, Lin et al. [11,12] proposed RetinaNet using the focal loss function, which uses the combination of a residual network (ResNet) and an FPN to output feature maps and predicts the bounding boxes and categories through two sub-networks. This network effectively suppresses the problem of class imbalance, but its network structure is relatively complex, and the inference speed is slow, making it difficult to meet the requirements of real-time inference. In 2018, Law et al. [13] proposed a model, CornerNet, that does not rely on an anchor box prior for prediction. This model uses the hourglass network as the backbone and predicts the upper left corner and lower right corner of the bounding box through two prediction branches. This anchor-free method reduces the selection of hyperparameters required by the model, but this method performs poorly in small objects and complex backgrounds, suitable for a single scenario. In 2020, Chen et al. [14] proposed a SAANet network model structure. Through sparse convolutional neural network learning of point cloud features, it uses a 2D convolutional neural network similar to ResNet to extract image features. Then, the spatial adaptive alignment (SAA) module automatically discovers complementary information between point cloud and image features, aligns the point cloud features and image features, and performs feature fusion. This method shows very fast inference speed and high average accuracy on the KITTI dataset. However, the prediction accuracy of this model significantly decreases on images with significant illumination changes.

In 2021, Cai et al. [15] proposed a single-stage object detection framework based on YOLOv4, with the backbone being CSPDarkNet53_dcn(P) and improving the last output layer by replacing it with deformable convolution. A new feature fusion module, PAN++, was designed, using five scales of detection layers, to improve the detection accuracy for small objects, but this model has poor adaptability in dynamic and complex scenes. In 2023, Wang et al. [16] proposed YOLOv7, introducing Efficient Layer Aggregation Network (ELAN) and Extended Efficient Layer Aggregation Networks (E-ELAN) as a new architecture design, significantly enhancing the feature learning ability and gradient flow of the network, effectively improving the detection performance of multi-scale objects. At the same time, the dynamic head module and improved label allocation strategy further optimize the training process, accelerating convergence speed while improving detection accuracy. Compared to YOLOv4, this model significantly improves the ability to handle complex environments. In dynamic video streams, although YOLOv7 has excellent real-time performance, it may still have false detections or missed detections for fast-moving or partially occluded objects.

These limitations indicate that convolution-based detectors still struggle to effectively capture long-range dependencies and global contextual relationships. Traditional convolutional networks rely on local receptive fields and stacked convolutional layers for feature aggregation, which, although computationally efficient, have disadvantages in long-range dependency modeling and global semantic relationship capture. They are prone to missed detections and category confusion when dealing with distant small objects, dense objects, and complex background textures. To address this deficiency, Transformer architectures based on self-attention mechanisms can explicitly capture cross-regional relationships through global token interaction, demonstrating stronger global expression capabilities in object detection. Representative examples include DETR and its subsequent improved series [17,18,19,20]. Among them, Deformable DETR reduces the computational cost of global attention to some extent and accelerates convergence through sparse deformable attention. However, such Transformer detectors still generally have high parameter counts and computational demands and are more sensitive to training strategies and data scale, which limits their direct deployment on resource-constrained edge platforms. Therefore, recent studies have attempted to integrate attention mechanisms into convolutional architectures in a lightweight manner. In recent years, Dai et al. [21] have explored integrating the attention mechanism in a lighter form into the CNN backbone, such as inserting multi-head self-attention or local window attention modules in the backbone or high-level features, and Li et al. [22] have adopted a hybrid architecture of convolution and attention to balance local details and global context. These methods typically enhance robustness in complex scenarios with relatively small additional overhead by reducing the resolution of attention computation, limiting the scope of attention, or introducing attention only in key layers.

Although significant progress has been made in the field of agricultural methods, there are still some problems:

1. Omissions and confusions caused by complex backgrounds and dense small objects. On rural roads, the scale of objects varies greatly, and the proportion of small objects at a distance is high. Moreover, the background texture (farmland vegetation, soil dust, debris) and occlusions are frequent. Although the YOLO model has a very fast inference speed, its accuracy drops significantly in high-dimensional and dense scenes. Detectors that rely solely on local convolutional representations are prone to omissions and confusion between similar categories.
2. The trade-off between global modeling capacity and lightweight deployment. The Transformer or self-attention mechanism can enhance long-range dependencies and global semantic associations. However, directly introducing global attention often leads to higher parameter counts and computational costs and is more sensitive to training strategies and data scale, making it difficult to meet the real-time deployment requirements at the edge. Therefore, the effective integration of the attention mechanism into a lightweight convolutional backbone at a relatively low cost to balance global semantics and local details is the key to improving the performance of rural road perception.
3. The multi-scale context and feature fusion still need to be optimized for agricultural scenarios. Multi-scale detection relies on effective context modeling and cross-layer feature fusion. However, under limited computational resources, the design of a lightweight multi-scale context module that works in synergy with the pyramid fusion structure, achieving an optimal balance between accuracy, complexity, and inference speed, remains a challenge.

## 3. Materials and Methods

The automatic navigation system for agricultural machinery [23] faces many challenges in the complex and dynamic rural road environment, such as strong background interference, variable lighting and weather conditions, easy object occlusion, and complex road conditions. Therefore, the demand for multi-object detection and obstacle perception is more urgent. In response to the above problems, this paper takes the rural road scene as the object and builds a multi-scale feature alignment and fusion detection model that adapts to complex environments to improve the object recognition ability of the system under low visibility, complex backgrounds, and dynamic interference conditions and provides reliable input for real-time obstacle avoidance and path planning.

**Figure 1:**
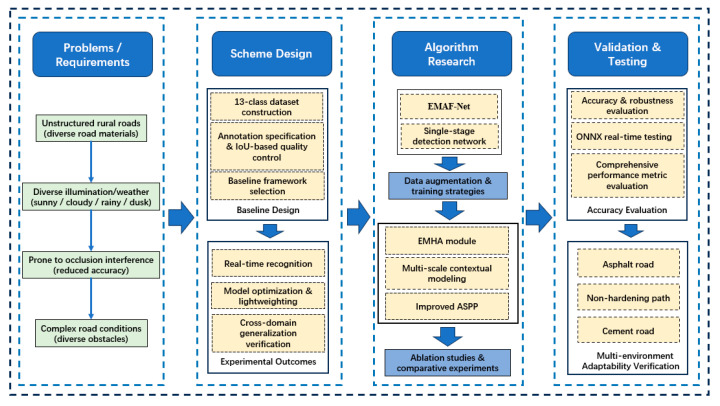
Overall technical framework for rural road object detection.

In the scheme design stage, this paper starts from data construction and task requirements, formulates the overall framework of object detection and multi-scale feature fusion, and combines the selection of the benchmark architecture and the constraints of lightweight deployment to establish a foundation for subsequent algorithm research. Figure 1 shows the overall architecture of this paper. In the algorithm research stage, a lightweight single-stage detection network, EMAF-Net, is proposed, featuring the EMHA backbone and a multi-scale fusion neck as its core. Strategies including data augmentation, optimized feature extraction, dilated/pyramid context modeling, integrated attention mechanisms, and improved loss functions are adopted to enhance detection accuracy and robustness in rural road scenarios. In the verification and testing stage, this paper systematically evaluates the proposed method from comprehensive indicators, such as detection accuracy and generalization ability, perception performance in complex rural road scenes, computational complexity, and real-time performance, and verifies its effectiveness and application potential on self-built and public datasets, further analyzing its adaptability and application potential in multiple environmental conditions.

### 3.1. Data Acquisition

This study has constructed a rural road object detection dataset that includes 13 object categories. By collecting high-resolution images, employing fine-grained annotations, and implementing strict quality control measures, it has addressed the issue of data scarcity in rural scenarios, providing a standardized benchmark for agricultural machinery intelligence.

#### 3.1.1. Data Collection Standards

The dataset references the formats of public datasets such as Cityscapes and BDD100K to collect representative sample images of rural roads. A GoPro HERO9 (Gropro Inc., San Mateo, CA, USA) single-lens visual motion camera is fixed in the center of the vehicle’s front windshield to simulate the driving view of agricultural machinery for data collection, with a resolution of 3840 × 2160 and a frame rate of 30 fps. Using this camera can avoid problems such as overexposure due to direct strong light, such as reflections from vehicle metal surfaces, and shadow occlusion, such as tree shadows blocking pedestrians. When the HDR mode of the camera is enabled, multi-frame synthesis can be used to retain high dynamic range details, and the ISO upper limit (≤400) can be set to suppress noise, thereby reducing the risk of blurred object boundaries. To address the issue of increased noise (reduced signal-to-noise ratio, SNR) and color temperature shift (enhanced warm tones) caused by low light, which leads to an increase in the missed detection rate of small objects (such as bicycles and traffic signs), the HyperSmooth 3.0+ low-light mode can be enabled. In low-light conditions, the frame rate is automatically reduced (e.g., from 30 fps to 24 fps), the exposure time is extended to reduce noise, and manual enhanced annotation of low-visibility objects is performed during the annotation stage.

**Figure 2:**
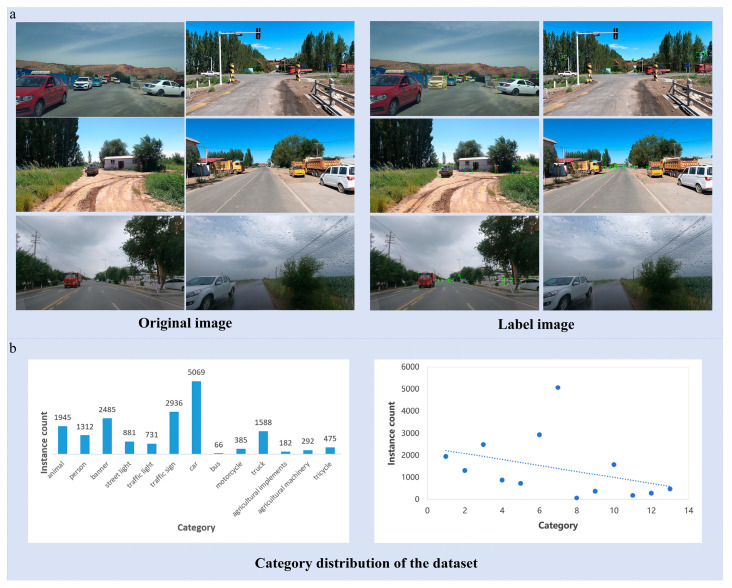
Overview of data collection: (**a**) an example of the collected data along with its annotations; (**b**) instance count for each category in the dataset (left: bar chart; right: scatter plot, where blue dots indicate the instance count for each category and the dashed line represents the linear trend of instance distribution).

The data collection areas are Qitai County and Hutubi County in Changji Prefecture, and the 142nd and 143rd Regiments in Shihezi City, Xinjiang, China. These areas are mainly selected because Qitai County of Changji Prefecture contains plain farmland, hilly pasture and the Gobi transition zone. Hutubi County contains the northern foot of the Tianshan gentle slope and the oasis agricultural area. Shihezi Regimental Farm is dominated by large-scale farmland grid roads, and this diverse terrain can fully cover the unstructured (field dirt roads) and semi-structured (cement roads, sand and stone roads) scenes common in agricultural machinery operations. Secondly, the above area is a mixed area of agriculture and animal husbandry. Road users include agricultural machinery (tractors, harvesters), livestock vehicles, cycling herders, free-range livestock (cattle, sheep), etc. The typical challenges of mixed traffic and frequent dynamic obstacles on rural roads are highly reproduced. Data collection covers asphalt roads, cement roads, and non-hardening paths, as well as multi-time scenes such as daytime (sunny, cloudy, overcast, rainy) and nighttime, ensuring light intensities range from 2000 to 100,000 Lux (daytime) and 50 to 500 Lux (nighttime). Data acquisition was performed at a steady speed of 30 ± 2 km/h, resulting in 6 h of continuous video. After frame extraction and initial screening, 4771 images were retained, totaling 18,347 instances, covering dynamic objects (person, animal, agricultural machinery, etc.) and static facilities (traffic light, traffic sign, banner, etc.). Based on the statistics of all ground-truth bounding boxes in the dataset, the minimum bounding box size is approximately 2.26 × 3.17 pixels (area ≈ 9.10 pixels^2^), while the maximum bounding box area reaches approximately 4.97 × 10^6^ pixels^2^. Relative to the original image resolution (3840 × 2160), the minimum and maximum bounding box areas correspond to approximately 0.00011% and 59.96% of the image area, respectively. This wide range indicates that the dataset contains objects with significant scale variation. The presence of extremely small objects further increases the difficulty of object detection in rural road scenes. The image acquisition situation is shown in Figure 2.

In order to comprehensively compare the differences and advantages between the rural road image dataset collected in this experiment and the existing mainstream road object detection datasets, a statistical comparison is conducted from five dimensions: lighting conditions, road surface characteristics, weather diversity, number of categories, and image resolution. The compared datasets notably include BDD100K, D2-City, Caltech Pedestrian, CityPersons, and NightOwls, as shown in Table 1.

As can be seen from the table, most of the existing datasets focus on urban roads, such as CityPersons, D2-City, and Caltech Pedestrian, lacking coverage of rural or unstructured roads. Moreover, the distribution of night and severe weather scenes is unbalanced; for example, there is almost no night or rainy data in the Caltech and CityPersons datasets. Furthermore, these datasets generally have low image resolution (720p or 1080p), which limits the accuracy of small object detection. Compared with other datasets, the rural road object detection dataset constructed in this experiment has obvious advantages in the following aspects:

1. More comprehensive scene coverage: The rural road image dataset collected in this experiment contains both urban road and rural road scenes, which makes up for the lack of existing datasets for non-urban areas and is especially suitable for applications such as rural traffic, agricultural machinery supervision, and automatic driving in remote areas.
2. Richer illumination and weather conditions: the images collected in this experiment completely cover day and night scenes, and cover typical meteorological conditions such as sunny days, cloudy days, and rainy days. This diversity of background can improve the robustness of the model in complex environments.
3. Diverse pavement types: the collected pavement features include many typical rural road types such as asphalt roads, cement roads, and unhardened roads, which are closer to the actual application scenarios.
4. Diverse categories: it supports 13 categories of object detection, which is more abundant than Caltech and BDD100K. The labels of our dataset include categories such as animals, agricultural machinery, and agricultural tools, which are basically not labeled in public urban street view datasets.
5. Higher image resolution: Ultra HD images with a resolution of 3840 × 2160 (4K) are adopted, providing higher spatial resolution than most mainstream datasets and improving the detection accuracy of small and distant objects, thereby creating a more realistic and challenging environment for multi-object detection and downstream tasks.

#### 3.1.2. Data Annotation

The data labeling work is based on LabelImg, and there are 13 categories in total: animal, person, banner, street light, traffic light, traffic sign, car, bus, motorcycle, truck, agricultural implements, agricultural machinery and tricycle. The xml standard format is used to store the bounding box category labels. Two groups of annotators (group A and group B) were set to annotate the bounding box of each image separately, and the different areas were repeatedly modified after expert review to ensure the quality of data annotation. In addition, referring to the multi-threshold evaluation standard of 0.5–0.95 generally used in CVPR data annotation, standard datasets generally set IoU ≥ 0.5 to be considered as a true positive. Therefore, a more stringent evaluation criterion is adopted, with the IoU consistency threshold set to ≥0.75. This requirement ensures that the bounding boxes annotated by Group A and Group B exhibit a high degree of overlap; otherwise, a second round of annotation refinement is performed until the images meet the predefined annotation standards. The annotation quality ablation experiment also demonstrated that when the IoU threshold is increased from 0.5 to 0.75, the mAP@0.5:0.95 of the agricultural machinery detection model is increased by about 2.89% and the False Negative Rate (FNR) is reduced by 2.4%. This result demonstrates that the quality of manually labeled data has a significant effect on the accuracy of model detection of complex samples.

In order to ensure the independence of the geographic region, this study employs a Hash Bucketing strategy (Algorithm 1). The name of the collection region is mapped to a 128-bit hash value through the MD5 Hash Function, the first 8 characters are intercepted and converted to an integer, and normalized to the interval value of [0, 1) by modulo operation. It is then split proportionally into training (80%), validation (10%), and test (10%) sets. This method ensures that the data of the same geographical area are assigned to the same data subset (training set, validation set or test set) and avoids the data of the same area appearing in multiple subsets at the same time, which effectively prevents the model evaluation bias caused by data distribution overlap, thereby improving the reliability of model evaluation.

**Algorithm 1**: Dataset splitting based on geographic region hashing (hash bucketing)Input: region: string   // Geographic region nameOutput: subset: {train, val, test} 1: // Step 1: Generate normalized hash value2: hash_str ← MD5(region)                       // Compute MD5 hash of region3: hash_hex ← Substring(hash_str, 0, 8)   // Extract first 8 hexadecimal characters4: hash_int ← HexToInt(hash_hex)           // Convert hexadecimal substring to integer5: hash_mod ← hash_int MOD 10,000     // Limit value range via modulo operation6: hash_ratio ← hash_mod/10,000.0       // Normalize to [0, 1) 7: // Step 2: Assign subset based on ratio8: if hash_ratio < 0.8 then9:          subset ← “train”          // 80% for training set10: else if hash_ratio < 0.9 then11:          subset ← “val”          // 10% for validation set12: else13:          subset ← “test”          // 10% for testing set14: end if

#### 3.1.3. Data Preprocessing and Augmentation

Frames were extracted from the original video using FFmpeg at 1 frame per second. After that, the extracted frames were filtered to eliminate the blurred frames with a Laplacian gradient value of less than 200. The Laplacian gradient value is used to measure the sharpness of the image, and a smaller value indicates a more blurred image. Invalid frames with occlusion area greater than or equal to 50% were removed.

After frame extraction and filtering, data augmentation was applied to improve the diversity of the training samples. The Albumentations [24] library was used to perform two types of augmentation [25]: diversified processing of the original images and joint augmentation of the images and bounding boxes. The specific augmentation operations for the original images include random horizontal flipping with a probability of 0.5, rotating the images by ±15°, adjusting brightness by ±20% and saturation by ±30%, injecting Gaussian noise with a standard deviation (σ) ranging from 0.01 to 0.05, and adding random occlusion blocks that account for 10–20% of the image area to simulate real-world interferences. The specific operations of the joint enhancement of the image and the bounding box include: at the same time of the above enhancement operation on the image, combined with the bounding box information in the image, the corresponding transformation is performed on the bounding box synchronously to ensure the consistency of the enhanced image and the bounding box annotation information, so as to provide data more suitable for the actual scene for model training.

**Figure 3:**
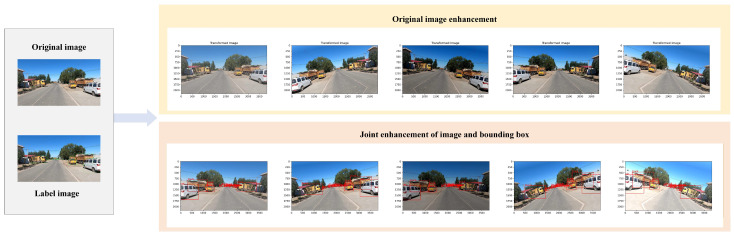
Comparison of results between two image enhancement methods.

To visually show the effects of these two enhancement methods, Figure 3 shows the original image on the left. In the middle are multiple results of data augmentation on the original image, which highlight the visual changes after only enhancing the image itself, without the detection box. On the right, the image and the corresponding bounding box are augmented synchronously. Through this comparison, the influence of different enhancement methods on the image and the relationship between the image and the bounding box can be clearly understood, which provides an intuitive basis for understanding the process and effect of data enhancement. Compared with image-only augmentation, the joint augmentation strategy maintains the spatial consistency between objects and their annotations. In this way, the model can learn more relevant and accurate features in the training process, so as to show stronger adaptability and stability in the face of complex and variable actual scenes, and then effectively improve the detection accuracy and generalization ability of the model. At the same time, due to the synchronization enhancement of the image and the bounding box, the annotation deviation that may be caused by the sequential processing is avoided, the consistency and reliability of the data are further guaranteed, and a higher-quality and more practical data basis is provided for model training.

After the above data augmentation process, this dataset presents the following unique characteristics. It provides a fine-grained object detection benchmark for rural road scenes in Xinjiang, China, including additional categories such as agricultural machinery and agricultural implements that frequently appear in unstructured rural environments. In order to control the problem of class imbalance, oversampling (for minority classes such as buses and agricultural implements) and undersampling (for majority classes such as cars and traffic signs) strategies are adopted to improve the detection performance of the model for each class of objects. The data covers multiple seasons (spring tillage, summer pipe, autumn harvest), multiple weather conditions and complex road conditions, which can provide a joint detection benchmark for dynamic obstacles (such as people, animals) and static facilities (traffic signs, traffic lights) for agricultural machinery automatic driving models. It promotes the robustness of the algorithm under low light, occlusion and multi-object interference. The dataset is managed by DVC version control, with metadata index (csv), MD5 verification files and technical documents to ensure traceability and reproducibility.

### 3.2. Object Detection Model Based on EMAF-Net

In order to build an efficient object detection model for an agricultural machinery automatic driving scene, a hybrid architecture model, EMAF-Net, is constructed based on the single-stage object detection algorithm. Combined with the architectural advantages of CNN and Transformer [26], based on the design idea of YOLOv4-P6, the joint module of convolution and multi-head self-attention was introduced to improve the efficiency of feature extraction, while taking into account the light weight of model parameters and global modeling ability. The real-time detection and recognition of 13 common object categories in rural road scenes was realized. YOLOv4-P6 was selected as the baseline architecture because it provides a good balance between detection accuracy, structural maturity, and computational efficiency for real-time detection tasks. Compared with earlier YOLO versions, YOLOv4 integrates several effective design strategies, such as CSPDarknet as the backbone network, PANet for multi-scale feature fusion, and various training optimization strategies, which make it a strong and stable baseline for further architectural improvements. For agricultural machinery automatic driving scenarios, real-time performance and deployment stability are important considerations. Therefore, a mature and computationally efficient architecture is more suitable as the foundation for model improvement. Based on the design idea of YOLOv4-P6, the proposed EMAF-Net introduces a joint module combining convolution and Multi-Head Self-Attention (MHSA) to enhance feature extraction capability while maintaining lightweight model parameters and global context modeling ability.

**Figure 4:**
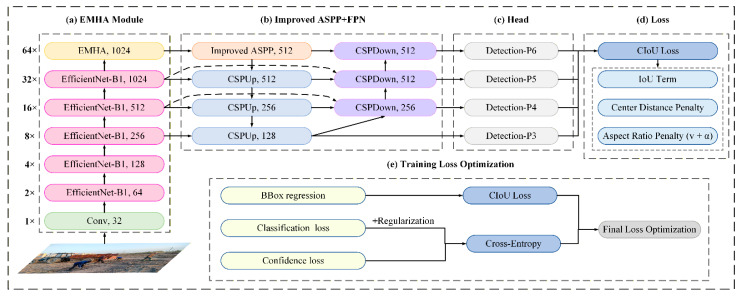
Schematic diagram of the basic architecture of EMAF-Net.

The network structure of the proposed model is shown in Figure 4, which mainly contains the following structures: backbone, neck, and head. Figure 4a shows the backbone part, which uses the lightweight model EfficientNet-B1 [27] that can better meet the deployment requirements of edge computing devices and combines multi-head self-attention to form the EMHA module. By replacing CSPDarknet in the original model and keeping the P6 structure unchanged, the computational efficiency and reasoning speed of the backbone network are significantly improved. The main function is to realize the feature extraction of the image by the model, and the specific details are described in detail in Section 3.2.1. Figure 4b shows the neck part. The Improved Atrous Spatial Pyramid Pooling (Improved ASPP) layer is combined with Feature Pyramid Networks (FPNs). The purpose of this is to enhance the multi-scale receptive field of the model and improve the detection ability of complex scenes and small objects, and the added spatial pyramid pooling can further improve the robustness of different-scale objects. FPN can also fuse different levels of feature maps, which is explained in detail in Section 3.2.2. Figure 4c illustrates the detection head, where the YOLO head is employed for prediction, ensuring high inference speed and end-to-end optimization capability. The details are described in Section 3.2.3. Figure 4d represents the loss function, which adopts the Complete IoU Loss (CIoU Loss) to improve the localization accuracy and classification performance of the bounding box. The details of the design and optimization of the loss function will be explained in Section 3.2.4. Figure 4e shows the method used in the training process, which will be described in detail in the Experiment Section 4.2.

In addition, this study also uses the Albumentations method for data augmentation, the K-means algorithm to cluster the prior of the object box in the training set, and these prior boxes on the feature map after multi-scale feature fusion to predict and classify the object box.

#### 3.2.1. EMHA Module

In the context of automatic navigation for agricultural machinery, the working environment often features complex backgrounds, significant differences in object scales, and frequent dynamic changes. This imposes higher demands on the feature extraction capabilities and real-time performance of detection models. Based on this, this paper improves the structure design of the lightweight detection model YOLOv4-P6. The reasons for choosing P6 over P7 are mainly as follows: (1) P6 reduces the feature map resolution to only 1/64 while maintaining semantic information, which can better preserve spatial details and is suitable for detecting medium-sized objects commonly found in rural road scenes (such as pedestrians and vehicles). At the same time, high-resolution feature layers like P3 (stride = 8) and P4 (stride = 16) are retained, providing stronger detail capture capabilities for the majority of small and medium-sized objects (accounting for over 90%). (2) The feature map resolution of P7 is extremely low (approximately 5 × 5), which is only helpful for very large objects but offers no practical benefit for small objects. More importantly, to make P7 effective, the input image resolution usually needs to be increased, which will significantly increase the computational cost and is not conducive to real-time detection. Therefore, the P6 structure better meets the requirements of rural road scenes for the detection of small and medium-sized objects and real-time performance.

**Figure 5:**
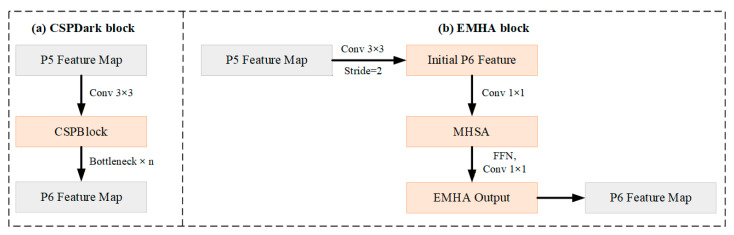
Comparison of the original model and the EMHA module backbone architecture.

However, although the traditional CSPDarkNet performs stably in YOLOv4-P6, its convolutional structure relies on local receptive fields, making it difficult to simultaneously consider global semantics and local details. As a result, it is prone to issues such as missed detections of small objects and category confusion in complex agricultural field scenarios. To address this, this paper replaces CSPDarkNet with an EMHA module composed of EfficientNet-B1 and a multi-head self-attention mechanism as the backbone network, as shown in Figure 5. Figure 5a illustrates the P5 → P6 feature extraction process of CSPDarkNet in the original YOLOv4-P6 model, while Figure 5b shows the structure in EMAF-Net where the P5 → P6 features are processed by the EMHA module.

EfficientNet-B1 achieves balanced expansion in depth, width, and resolution through a compound scaling strategy, which can enhance feature extraction efficiency while maintaining a low parameter count and computational cost, significantly reducing the power consumption and inference latency of embedded vehicle-mounted platforms. The multi-head self-attention structure, on the other hand, breaks through the limitations of local modeling by convolutional operations, enabling the capture of long-range dependencies and global semantic relationships across object regions in feature maps. This effectively mitigates the decline in detection performance caused by scale variations and perspective differences. The combination of these two modules enables the EMHA module to achieve a better balance between detection accuracy and computational efficiency, not only enhancing the model’s robustness in small object detection, complex background discrimination, and occlusion scenarios but also ensuring the real-time perception and decision-making requirements of agricultural machinery in rural road scenarios. Therefore, replacing CSPDarkNet with the EMHA module is reasonable and can provide more abundant and globally correlated input features for subsequent multi-scale feature fusion such as Improved ASPP and FPN, thereby laying a solid foundation for the overall improvement of detection performance.

The main contributions of this section are as follows: The first part involves selecting and improving the internal structure of EfficientNet, and the second part involves effectively combining convolution and self-attention mechanisms. EfficientNet is mainly composed of the Mobile Inverted Bottleneck Convolution (MBConv) module, which consists of the inverted bottleneck structure and the squeeze and excitation (SE) module based on the channel domain attention. It uses Swish (Self-Gate Activation Function) as the activation function, which has been proven to be more effective than the ReLU activation function on some challenging datasets. Compared to CSPDarkNet, EfficientNet reduces the computational cost by using a compound scaling method that balances the depth, width, and resolution dimensions. This improves the recognition accuracy.

In this paper, the global average pooling layer, fully connected layer, and classification layer at the end of the EfficientNet-B1 model are removed, leaving 7 groups of MBConv modules. Using the selection method of YOLO feature layers, the network layers with downsampling ratios of 8, 16, and 32 are selected as the feature maps extracted by the backbone network, corresponding to the 4th and 6th MBConv in the network and the output of the backbone network. However, due to the small dimension of the feature maps output by the MBConv, using them as feature layers will affect the representation ability of the subsequent model. To enable the model to have better fitting ability, a new feature layer combination with the same downsampling ratio is selected to obtain a more comprehensive feature representation. Considering that in each group of MBConv, the first 1 × 1 convolution amplifies the feature dimension, increasing the model’s feature representation, and this MBConv does not contain shortcut links, it will not cause feature loss. Therefore, the output of the first 1 × 1 convolution kernel in the 5th and 7th MBConv and the output of the backbone network are selected as the feature extraction results.

However, since the deep separable convolution essentially belongs to a local operator, the model has deficiencies in capturing long-range dependencies and global context relationships. To address this, this paper introduces the MHSA module after the key feature layers of the EfficientNet backbone. This module can model the global dependencies of features in different subspaces through parallel attention heads, thereby compensating for the deficiencies of MBConv in global semantic capture. By combining the efficient local representation of EfficientNet with the global modeling ability of MHSA, the improved backbone network EMHA module can generate more discriminative and robust multi-scale feature representations while remaining lightweight, providing a solid foundation for the object detection task in complex rural road scenarios and providing higher-quality input features for the subsequent neck network’s cross-scale feature fusion.

#### 3.2.2. Neck for Robust Multi-Scale and Small-Object Detection

In the neck, due to the large number of objects and diverse scale sizes leading to decreased detection accuracy, an Improved ASPP and bidirectional FPN feature fusion structure is adopted: on the high-level features, the Improved ASPP introduces multi-scale context to expand the receptive field; subsequently, based on the top-down fusion of FPN, a bottom-up feedback path (implemented by CSPUp/CSPDown fusion blocks) is introduced for the re-aggregation of multi-scale information, thereby enhancing scale robustness and small object detection capabilities. Different from the standard ASPP, which only relies on dilated convolution branches with different dilation rates, the Improved ASPP in this paper expands the effective receptive field through multiple branches of dilated convolution to capture sparse long-range context.

**Figure 6:**
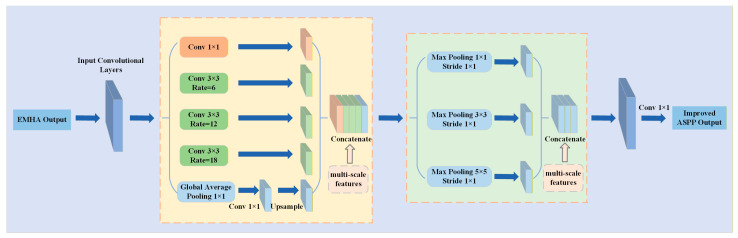
Improved ASPP model structure diagram.

At the same time, a pyramid pooling branch is introduced to supplement the aggregation ability of statistical information for different scale regions, and the outputs of each branch are concatenated in the channel dimension and compressed and re-organized using convolution to obtain a richer and more robust context representation with less additional computational overhead. By using the idea of spatial pyramid pooling, the maximum pooling layers with sizes of 1 × 1, 3 × 3, and 5 × 5 are used for multi-scale pooling, where the 3 × 3 and 5 × 5 pooling are used to introduce local context information of different scales, and the 1 × 1 pooling branch is equivalent to the identity mapping to retain the original feature information and stabilize the fusion. All pooling operations are set with a stride of 1 and padded to maintain the spatial size of the feature map, and then the outputs of each branch are concatenated in the channel dimension for fusion. By introducing a small amount of computation, the receptive field of the backbone network is further expanded, enabling the network to have better adaptability to the large objects occupying areas in the original image. This model aims to enable the backbone network to obtain richer feature information and improve the performance of the model when processing objects of different scales, using the output feature maps of the first pointwise convolution in MBConv5 and MBConv7 in the backbone network and the fused output feature map of the neck network as the input of differently sized feature maps of the head layer. By making such a selection, a comprehensive analysis of the depth and spatial resolution distribution of the feature layers can be conducted, enabling the fusion of features at different levels. The structure of the Improved ASPP is shown in Figure 6.

#### 3.2.3. Object Detection Head

The head is the module that outputs the final detection results. In this paper, the YOLO head is used to output the final bounding boxes and categories. It mainly calculates the loss by comparing the predicted values with the true values, reshapes the data format, and finally extracts the features to output the feature map. The head of this model is selected because of its simple and efficient structure, which can ensure the real-time inference speed of the model. It is composed of only two 1 × 1 and 3 × 3 convolution kernels, and makes the final prediction on the 4 feature maps output by the neck layer. Each prediction result is a tensor in the channel dimension, which can be expressed as:

(1) $$3\times \left(5+C\right)$$

C represents the number of object categories, and the multiple 3 indicates that each prediction corresponds to three prior boxes of different sizes. The number 5 represents the four coordinates of the box and one confidence level. The 5+C represents the dimension, which is the number of attributes for each prediction. It saves the center point coordinates, the length and width of each box, the probability of the existence of an object, and the probability that the object in the box belongs to each category. The predicted length and width are scaling factors and need to be multiplied by the size of the prior box to obtain the final predicted size.

To obtain this prior information, the Intersection over Union (IoU) is used as the distance calculation formula between sample points and centroids. The K-means algorithm is applied to cluster all the object boxes in the training set, resulting in 12 prior boxes of different sizes. Finally, the model’s prediction results are multiplied by their corresponding prior boxes to obtain the predicted object boxes. Non-Maximum Suppression (NMS) is used to filter all the predicted object boxes. Based on the confidence level and IoU threshold of the predicted boxes, the boxes with high overlap are filtered out, and the box with the highest confidence level is retained as the detection result.

#### 3.2.4. Loss Function

During the model training process, the CIoU loss function is used to calculate the localization error between the predicted boxes and the real boxes. Different from the traditional MSE (Mean Square Error) loss, the CIoU loss function takes into account factors such as the overlapping area of the boxes, the distance between the centers, and the aspect ratio of the boxes simultaneously, thereby solving the problems of gradient explosion and dissipation that may occur during the gradient propagation process of the MSE loss. The MSE loss only relies on the positional and size differences between the predicted boxes and the real boxes, and its formula is as follows:

(2) $$L_{Y}=\sum_{i=0}^{S^{2}}\sum_{j=0}^{B}l_{ij}^{abj}\left(2-w_{i}\times h_{i}\right)\left[{\left(x_{i}-{\hat{x}}_{i}\right)}^{2}+{\left(y_{i}-{\hat{y}}_{i}\right)}^{2}+{\left(\sqrt{w_{i}}-\sqrt{{\hat{w}}_{i}}\right)}^{2}+{\left(\sqrt{h_{i}}-\sqrt{{\hat{h}}_{i}}\right)}^{2}\right]$$

$x_{i}$, $y_{i}$, $w_{i}$, $h_{i}$ represent the center point coordinates, width and height of the predicted bounding box. ${\hat{x}}_{i}$, ${\hat{y}}_{i}$, ${\hat{w}}_{i}$, ${\hat{h}}_{i}$ represent the center point coordinates, width and height of the actual bounding box. S and B represent the size of the grid and the number of prior boxes, respectively, and $l_{ij}^{abj}$ indicates whether the current grid contains the object.

However, MSE is prone to the problems of gradient explosion and gradient dissipation. In the early training stage, due to the large gap between the true value and the predicted value, the loss value will tend to infinity, causing the model to fail to train. In the later training stage, the loss function fluctuates around a stable value, making it difficult for the model to converge to higher accuracy, and it is prone to fall into a local optimal solution. To improve localization accuracy and stabilize the training process, the CIoU loss function is adopted as the bounding box regression loss. The CIoU loss not only includes the traditional IoU calculation but also introduces additional constraints, such as center point distance and aspect ratio difference, to more comprehensively measure the error between the predicted box and the true box. The formula of this loss function is:

(3) $$L_{C}=1-IoU+R_{CIoU}$$

(4) $$R_{CIoU}=\frac{\rho^{2}\left(b,b^{gt}\right)}{c^{2}}+\alpha v$$

(5) $$v=\frac{4}{\pi^{2}}{\left(arc\tan\frac{w^{gt}}{h^{gt}}-arc\tan\frac{w}{h}\right)}^{2}$$

(6) $$\alpha =\frac{v}{\left(1-IoU\right)+v}$$

IoU represents the traditional intersection-over-union calculation for the predicted box and the real box. $\rho^{2}\left(b,b^{gt}\right)$ represents the Euclidean distance between the center point of the real box and the center point of the predicted box. c represents the diagonal length of the smallest rectangle formed by the real box and the predicted box. v represents the difference between the aspect ratio of the real box and the predicted box. α is a balance parameter. When the intersection over union of the real box and the predicted box is small, this parameter reduces the weight of v to make the model focus more on the regression of the center point and the dimensions of the bounding box.

It can be seen that compared with MSE, CIoU considers more factors and can drive the model to predict more accurate and closer-to-object predicted boxes. However, in the gradient backpropagation, the v calculated in Formula (5) is prone to gradient explosion and gradient dissipation problems. Especially in Formulas (7) and (8), $w^{2}+h^{2}$ is located in the denominator, directly causing instability in the gradient value. Therefore, when calculating the gradient value, Formulas (7) and (8) are simultaneously multiplied by $w^{2}+h^{2}$ to ensure the stable backpropagation of the gradient. The derivative formula of v with respect to w and h is:

(7) $$\frac{dv}{dw}=\frac{8}{\pi^{2}}(arc\tan\frac{w^{gt}}{h^{gt}}-arc\tan\frac{w}{h})(\frac{h}{w^{2}+h^{2}})$$

(8) $$\frac{dv}{dh}=-\frac{8}{\pi^{2}}(arc\tan\frac{w^{gt}}{h^{gt}}-arc\tan\frac{w}{h})(\frac{h}{w^{2}+h^{2}})$$

In this way, it is possible to ensure that no instability occurs during the gradient calculation, thereby making the model training more stable and efficient.

## 4. Results

### 4.1. Experimental Data

The rural road dataset consists of 4771 images captured by a monocular vision camera in a complex rural road scenario, belonging to 13 categories and containing 18,347 instances. Among them, 3817 images are used for training, 476 images are used for result validation, and the remaining 478 images are used for testing. The images in this dataset were collected under diverse weather conditions, varying illumination levels, and different road surface materials. In addition, the scenes contain complex backgrounds and frequent occlusions, which increase the diversity and challenge of the dataset and help improve the robustness and generalization ability of the model. All images were manually annotated to ensure the accuracy and reliability of the experimental data. In addition, the BDD100K dataset, a large-scale benchmark for autonomous driving research, was used to further evaluate the detection performance. This dataset contains a total of 100,000 images, 10 categories, 70,000 training images, 10,000 validation images, and 20,000 test images. This dataset was chosen because the BDD100K dataset has a high similarity in scene composition with the rural road dataset constructed in this paper, covering various weather, lighting, and road types, and can reflect typical urban and suburban traffic scenarios. Pre-training on this dataset not only enables the model to learn more robust feature representations on a large and diverse set of samples but also significantly improves the convergence speed and detection accuracy when transferred to the rural road scenario, thereby enhancing the model’s adaptability to specific scenarios. Moreover, introducing it into the validation phase can effectively evaluate the model’s performance in different geographical regions, road structures, and imaging conditions, thereby testing the model’s cross-domain robustness and generalization ability.

### 4.2. Experimental Environment and Training Settings

All experiments were conducted on a workstation equipped with an NVIDIA RTX 4090 GPU (NVIDIA Corporation, Santa Clara, CA, USA) and 24 GB RAM. The models were implemented in PyTorch 1.13 with CUDA 11.7 under Ubuntu 20.04.

In the experiment, the K-means clustering algorithm was used to cluster the width and height of the object boxes in the training set, resulting in anchors, which were used for subsequent training. Bounding box regression was employed to align the predicted object windows closer to the ground truth windows. In the coordinate regression of detecting the object box of the head, the Sigmoid output of the coordinate center point was amplified by 1.05 times to better position the center point closer to the grid edge of the object. The entire training process was divided into two stages. The first stage was to freeze the backbone. Firstly, an Adam optimizer with high computational efficiency and low memory requirements was used, with a learning rate of 0.001, and 50 epochs of training were conducted. Since the Adam optimizer might not converge, the second stage unfroze the entire network and trained for 250 epochs using an SGD optimizer with a learning rate of 0.0001 and a momentum of 0.9. Gradient updates were performed for each sample during each update to avoid redundant computations and not miss the global optimal solution. The calculation process is shown in Formulas (9) and (10).

(9) $$\eta_{t}=\alpha \cdot g_{t}$$

$\eta_{t}$ represents the current moment’s descent gradient, α is the initial learning rate, and $g_{t}$ is the gradient calculated for the object function with respect to the current parameters, that is, $g_{t}=m_{t}=\varphi \left(g_{1},g_{2},\ldots ,g_{t}\right)$.

(10) $$\omega_{t+1}=\omega_{t}-\eta_{t}$$

$\omega_{t+1}$ represents the updated calculation of the descent gradient, while $\omega_{t}$ refers to the parameters to be optimized in the t-th epoch, and $\eta_{t}$ represents the descent gradient at the current moment.

**Figure 7:**
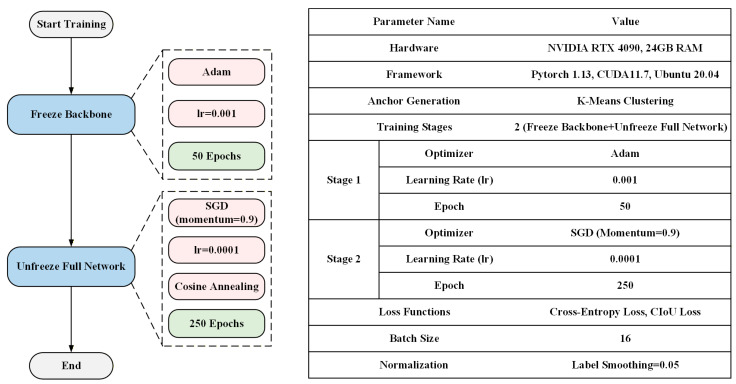
Model training process and parameter setup diagram.

Since the SGD optimizer does not utilize second-order momentum and the learning rate is constant, a cosine annealing strategy needs to be adopted for the learning rate to ensure a monotonically decreasing trend, eventually reaching 0, enabling the model to converge. The loss function uses the cross-entropy loss function as the loss function for the object class and the object box confidence, and the CIoU loss as the loss function for the regression of the object box coordinates. During training, the batch size of EMAF-Net is set to 16 based on the GPU’s available memory. The regularization method employs a label smoothing value of 0.05 to alleviate the problem caused by class imbalance. The model training process and parameter settings are shown in Figure 7.

During the training process, the performance of the model on the test set is output every 10 epochs. Firstly, a set of hyperparameters for the model is determined. The model is trained on the training set, and the model with the smallest loss function is selected. The selected optimal function is evaluated on the validation set, and the search for the specified hyperparameter combinations is continued until it is completed. The model with the smallest error on the validation set is selected, and the training set and validation set are combined as the overall training model to find the optimal function. This paper uses the criterion of whether the total loss has decreased as the standard. After each round of training, models with a decreased loss are saved, and otherwise, no saving is performed. Finally, the generalization ability of the optimal function on the test set is evaluated.

### 4.3. Evaluation Metrics

In object detection tasks, the average precision at an IoU threshold of 0.5 (mAP@0.5) and the average precision averaged over IoU thresholds from 0.5 to 0.95 (mAP@0.5:0.95) are adopted as the primary evaluation metrics to measure detection performance under single and multiple IoU criteria. The higher the mAP@0.5, the better the model’s detection accuracy for the objects. mAP@0.5:0.95 reflects the model’s detection capabilities across different scales and strict IoU levels. FNR is specifically introduced in the ablation experiment to analyze the impact of annotation thresholds on detection performance. It represents the proportion of true objects that the model fails to detect among all ground-truth objects. The smaller the FNR, the better. In addition, the GFLOPs of the model, which represents the number of floating-point arithmetic operations it can perform per second, is used to measure the computational complexity of the model. The fewer GFLOPs, the lighter the model is and the lower the computational resource requirements. The FPS (frames per second) of the model, which represents the number of image data frames it can process per second, is used to measure the real-time processing capability of the model. The higher the FPS, the better the model’s running efficiency and the more suitable it is for real-time scenarios.

### 4.4. Experimental Results

#### 4.4.1. Experimental Results of the Rural Road Dataset

**Figure 8:**
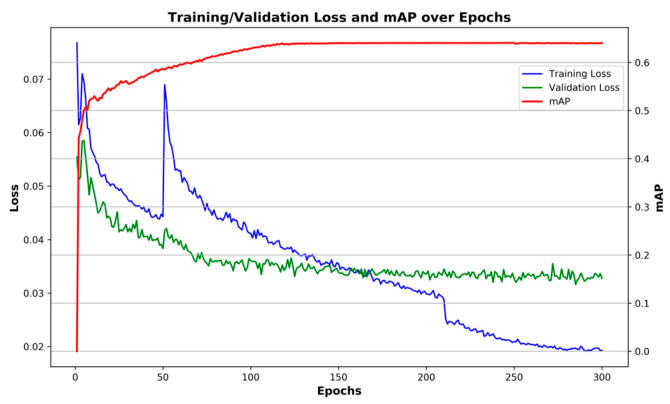
Trends of model training loss, validation loss, and mAP.

Figure 8 shows the curves of training and validation loss functions of EMAF-Net after 300 rounds of training on our self-built rural road object detection dataset, represented by blue and green lines, respectively, and the gradually increasing trend of mAP is indicated by the red line. From the figure, it can be seen that the model is divided into two stages during the entire training process: The first stage freezes the backbone and trains for 50 rounds using the Adam optimizer. As the epochs increase, the training loss and validation loss decrease significantly, demonstrating the model’s initial rapid convergence characteristic. The second stage unfreezes the entire network and trains for the next 250 rounds. The optimizer is switched to SGD and the learning rate is reduced using the cosine annealing strategy. It can be observed that the loss function of the model slows down more gradually in the second stage, and the validation loss stabilizes at around 250–300 epochs, indicating that the model has good generalization performance. EMAF-Net can maintain stable convergence over a long training period. Meanwhile, mAP gradually increases from the initial 0 and stabilizes at around 0.64. This indicates that EMAF-Net can effectively detect objects in rural road scenes through long-term training and optimization and maintain high detection performance even under strict IoU thresholds.

**Figure 9:**
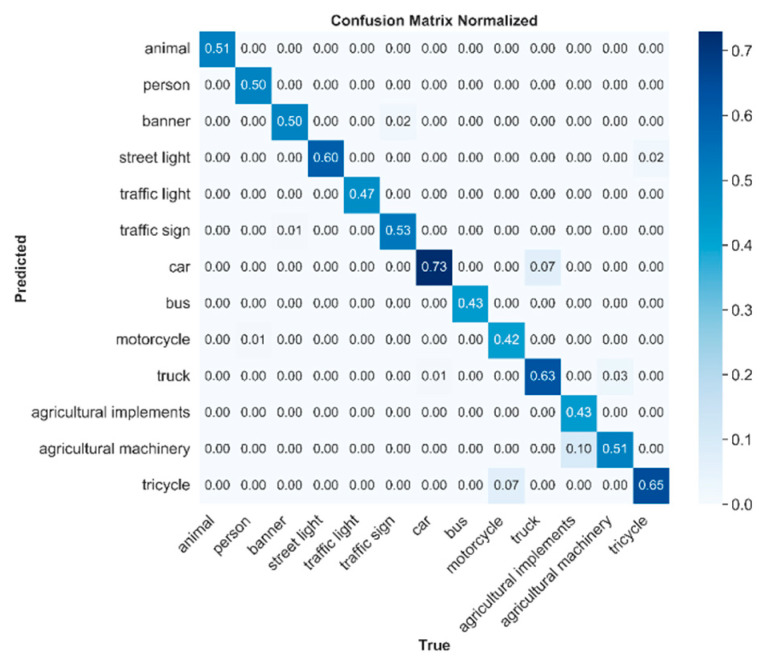
Confusion matrices for each category and correlation analysis results.

Figure 9 shows the confusion matrix of EMAF-Net on the rural road object detection dataset. Each element in the confusion matrix represents the normalized ratio between the true class and the predicted class. Values closer to the diagonal indicate higher classification accuracy of the model, while values off the diagonal reflect the degree of confusion between classes. From the figure, it can be seen that in scenarios with complex environments and large variations in object scales, EMAF-Net demonstrates a high accuracy rate in detecting multiple object categories. For example, the proportion of correct classification for the large-scale category car reaches 0.73, which is the highest among all categories. The correct classification proportions for tricycle and agricultural machinery are also 0.65 and 0.51, respectively. Moreover, the correct detection accuracy rate of the small-scale category street light by the model is 0.60, and the correct classification rate for traffic signs is 0.53. This fully demonstrates the model’s ability to detect complex and diverse objects. However, the figure also reveals that the model still has a certain degree of confusion for some categories. For instance, the truck category achieved a correct classification rate of 0.63, but some samples were still wrongly detected as a car (with a proportion of 0.07), while some samples of the motorcycle category were mistakenly identified as a tricycle (with a proportion of 0.07). This is because these object categories have similar shapes and appearances, and the front-end appearance of trucks and cars in rural road scenes is very similar, making them easy to misidentify. Some motorcycles and tricycles have a very small amount of confusion due to the frequent presence of riders blocking the details of the vehicle body, and their appearances are slightly different from each other from a frontal perspective, so most of them can still be correctly distinguished. Other categories do not have obvious confusion and can be correctly and undistortedly identified.

Overall, the confusion matrix results of EMAF-Net indicate that the model has a high accuracy rate in object detection in rural road scenes, accompanied by a small amount of misclassification between categories. In addition, missed detections may still occur in cases where objects are partially occluded. For example, agricultural implements attached to machinery or pedestrians riding on agricultural vehicles may be partially hidden by the vehicle body, making their visual features incomplete. Such occlusion reduces the discriminative features available to the detector and may lead to missed detections in some complex scenes. This issue is common in real rural traffic environments and remains a challenging problem for object detection models.

**Figure 10:**
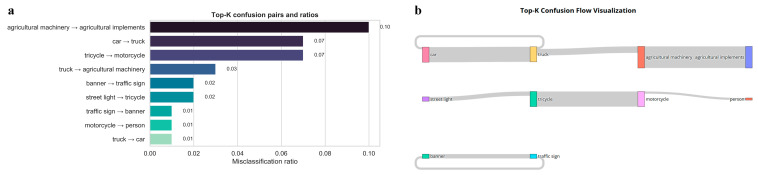
Major confusion pair analysis and confusion flow visualization: (**a**) Top-K confusion pairs and ratios; (**b**) Top-K confusion flow visualization.

Figure 10 shows the visualization of the main category confusion pairs and their flow directions on the rural road dataset by EMAF-Net. In Figure 10a, the Top-K confused category pairs and the corresponding confusion ratios are presented. Figure 10b presents the main confusion flow from the true category to the predicted category in a bar graph format. The thicker the connection line, the higher the confusion ratio. Overall, the Top-K analysis and the bar graph results are consistent with the confusion matrix conclusion in Figure 9; EMAF-Net can achieve stable discrimination for most categories in complex rural road scenarios.

To evaluate the detection performance of EMAF-Net, comparative experiments were conducted using three representative models: YOLOv7, Deformable DETR, and YOLOv11 [28]. These three models were chosen because YOLOv7 is a classic lightweight object detection model based on convolutional neural networks, with its structure design demonstrating highly optimized detection efficiency and fast inference capabilities, and it is representative in resource-constrained scenarios. Deformable DETR represents the object detection method based on the Transformer structure, which demonstrates advanced theoretical advantages in complex multi-object detection scenarios through the use of attention mechanisms and deformable convolutions but has a relatively high computational complexity. YOLOv11 is an improved version of the YOLO series, which further enhances the detection performance through parameter optimization and multi-scale feature extraction. These three models are representative in multiple dimensions and from a historical perspective and can fully reflect the improvement value of EMAF-Net in terms of detection performance, lightweighting, and real-time capabilities.

Table 2 presents the training results of all the selected object detection models on the self-built rural road dataset. To ensure fair comparison, all models were trained for 300 rounds to ensure sufficient convergence. However, it is worth noting that Deformable DETR has converged around the 150th round in this task experiment. Subsequent iterations have had limited improvement in the metrics, and some metrics even slightly decreased due to overfitting. Therefore, the best results of this model are used as the final data for the comparative analysis in the table.

From the table, it can be seen that in terms of accuracy, our improved EMAF-Net model has demonstrated significant advantages in all indicators. The mAP@0.5 reached 64.05%, which was 2.11% higher than the YOLOv11 model, 8.53% higher than YOLOv7, and 13.15% higher than Deformable DETR. Additionally, in the stricter IoU threshold of mAP@0.5:0.95, the accuracy of EMAF-Net was 48.95%, which was 4.63% higher than YOLOv11, 7.56% higher than YOLOv7, and 18.45% higher than Deformable DETR. In terms of running efficiency, the params of EMAF-Net were only 18.3 M, which was 18.6 M less than YOLOv7, 1.8 M less than YOLOv11, and 21.7 M less than Deformable DETR, fully demonstrating the lightweight characteristic of the model. In terms of model computational complexity, the GFLOPs of EMAF-Net was 38.5, which was 66.2% less than YOLOv7, 29.5% less than YOLOv11, and 134.5% less than Deformable DETR, further illustrating the advantage of EMAF-Net in computational efficiency. Moreover, in terms of real-time performance, after exporting to ONNX format on the NVIDIA RTX 4090 GPU, the FPS of EMAF-Net reached 184.62, which was 51.78% higher than YOLOv11, 27.6% higher than YOLOv7, and 137.32% higher than Deformable DETR. It is worth noting that although high-end GPU platforms can achieve extremely high inference speeds, the computational capabilities of edge devices in practical application scenarios are often limited. To verify applicability on typical edge computing devices, video inference experiments were conducted using a representative NVIDIA 1650Ti GPU. The results show that EMAF-Net achieves an average inference speed of approximately 27 FPS (39 ms per frame) on the 1650Ti, meeting the real-time requirements for rural road scene perception. The exported ONNX model can be directly deployed to most edge devices or applications without additional processing, demonstrating excellent practical value. Combining detection performance and computational efficiency, EMAF-Net achieved a balance between accuracy and lightweighting. Overall, EMAF-Net performed excellently in terms of detection accuracy, model lightweighting, and real-time performance, especially in complex rural road scenarios with limited resources. The experimental results verified the effectiveness of the model improvement strategy.

**Figure 11:**
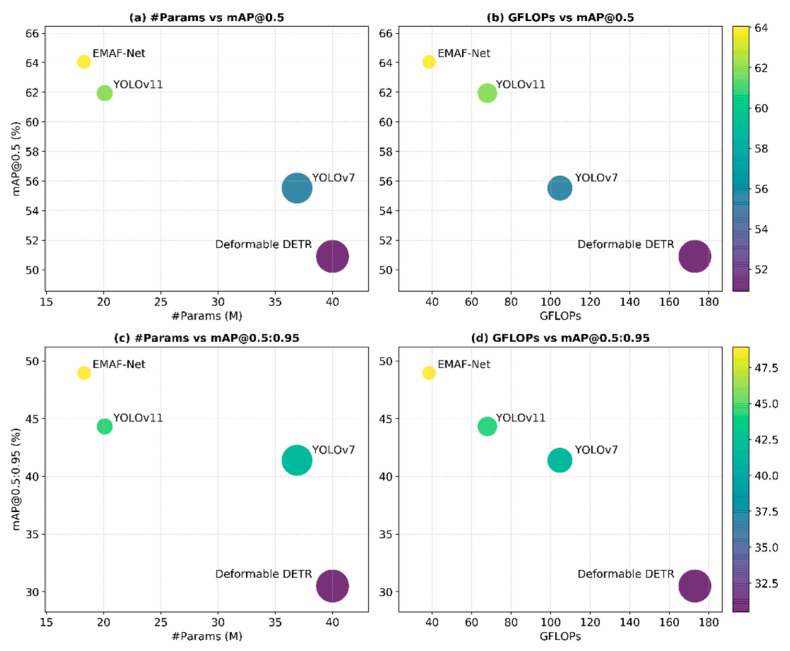
Accuracy and complexity trade-off of different detectors on the rural road dataset: (**a**) Comparison results of mAP@0.5 with changes in model parameters; (**b**) effect of GFLOPs on detection accuracy for mAP@0.5; (**c**) comparison results of mAP@0.5:0.95 with changes in model parameters; (**d**) effect of GFLOPs on detection accuracy for mAP@0.5:0.95.

To more intuitively reveal the trade-off relationship between different models in terms of accuracy and complexity, a visual analysis of the key indicators was further conducted. As shown in Figure 11, the relationship between model complexity and detection accuracy (mAP@0.5 and the stricter mAP@0.5:0.95) is illustrated from two perspectives: the number of parameters (Params) and computational complexity (GFLOPs). Here, the size of the bubbles is proportional to the model complexity: the first two subplots (a) and (c) are proportional to Params, and the last two subplots (b) and (d) are proportional to the computational cost (GFLOPs). Additionally, the color of the bubbles is used to represent the corresponding detection accuracy (mAP), with darker yellow indicating higher accuracy. Through this graph, one can more intuitively compare the accuracy performance of each method under different complexity constraints and verify the ability to achieve model lightweighting and efficient inference while improving detection accuracy, thereby more comprehensively demonstrating the advantages of EMAF-Net in terms of accuracy and efficiency.

**Figure 12:**
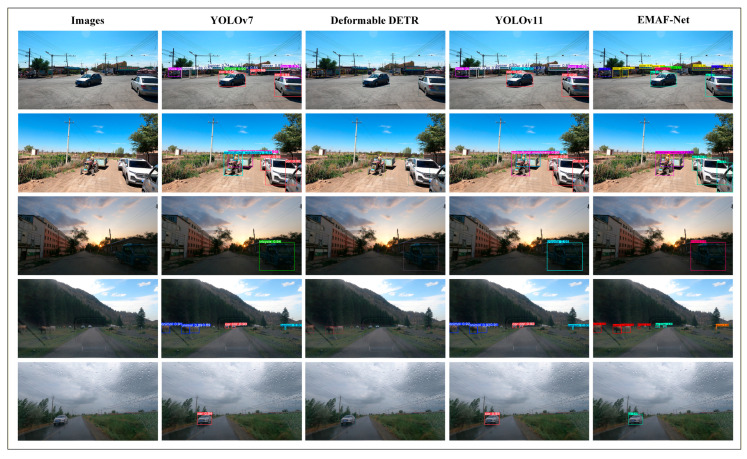
Visualization results of the rural road object detection model.

Based on the quantitative results and complexity trade-off analysis described above, qualitative comparisons of detection results among different models in complex rural road scenarios are further presented. Figure 12 shows the visual detection results of four models under different light intensities, different road surface materials, and different weather backgrounds. At the same time, in combination with the requirements of the detection task, it can comprehensively evaluate the object recognition performance of each model in different environments. This ranking is mainly based on the light intensity, presenting rural road scenarios with different road surface materials, including asphalt roads and non-hardening paths, transitioning gradually from bright daytime to dark rainy-weather scenes. The first picture in the first column shows a relatively wide intersection on the rural road, with abundant daylight, presenting a strong light condition. The road is paved with asphalt, and there are houses and power poles around, reflecting the relatively smooth but simple infrastructure of rural transportation. The second picture shows a typical rural road scene at the field edge, clearly showing agricultural machinery and small trucks. The road surface material is a non-hardening path, and the environment has sufficient light, with many crops and a lot of vegetation around, reflecting another type of rural road scene. The third picture shows the rural road scene at dusk, with the gradually weakening light changes adding complexity to the detection task. The fourth picture shows the mountain pasture scene with low-light conditions and a slippery road surface, with dense vegetation along the edges. This area has gradually decreasing light and an overall darker scene, able to reflect the unique transportation characteristics of remote rural areas. The fifth picture shows the cloudy environment of the road after rain and the wet road features, with the view affected by raindrops, presenting the complex and variable traffic environment and weather conditions of rural areas and also adding higher challenges to the detection task.

From the visual detection results, it can be seen that different models have significant differences in their ability to identify objects in complex environments. In high-light scenarios, YOLOv7 and YOLOv11 can capture common objects, such as cars and banners, with high confidence levels. However, they are insufficient in identifying complex objects like the small distant ones in the fourth row, such as animals and people. Deformable DETR has good robustness in object box positioning, and most detection boxes accurately select the object boundaries, but their confidence levels are generally low. Especially in low-light environments (such as rainy days and dusk scenes), their confidence levels significantly decrease, and they are prone to being affected by background interference, resulting in incorrect box selection phenomena. For example, in the second picture, the agricultural implements that were partially obscured were not detected, and the person category on the agricultural machinery was not detected either. In the fifth picture, the car was detected, but its confidence level was only 0.795, while other models were clearly above 0.9. In contrast, EMAF-Net demonstrates outstanding detection performance in complex scenarios, especially in the recognition of agricultural implements, banners, people, and animal categories. In the first picture, it can be clearly seen that our model has higher confidence levels than other models in all categories, especially in the banner category, where the confidence level is the highest at 0.9 in the YOLO series and 0.93 in our model. And the traffic light was only detected at 0.66 in YOLOv7, 0.712 in Deformable DETR, and only 0.58 in YOLOv11, while our model had a confidence level of 0.73. In the fourth mountainous low-light scene, EMAF-Net accurately detected multiple object categories such as car, animal, and person, and completed clear boundary box selection. Especially for the animal category, YOLOv7 and YOLOv11 missed some objects, while Deformable DETR had two cases of background misjudgment. In the fifth low-light, rainy environment, EMAF-Net still accurately captured distant vehicle objects and achieved the highest confidence level of 0.97.

By comparing the above results, it can be seen that EMAF-Net performs exceptionally well in complex categories and low-light environments, especially in the detection of distant and small objects. It is significantly superior to other models in capturing these categories. These experimental results further validate the robustness of EMAF-Net and its applicability in the complex rural road environment.

#### 4.4.2. Experimental Results of the BDD100k Dataset

To evaluate the generalization capability of the model on a public dataset, four representative detectors—YOLOv7, Deformable DETR, YOLOv11, and EMAF-Net—were selected for comparative experiments on the BDD100K dataset. The BDD100K dataset is a public dataset with rich driving scenarios, including various weather, lighting conditions, and complex road environments, and is an important benchmark for verifying the model’s generalization ability. Each model was uniformly trained for 150 epochs, and the results are shown in Table 3.

From the table, it can be seen that EMAF-Net performs the best on the BDD100K dataset. It demonstrates outstanding detection performance in key metrics such as mAP@0.5 and mAP@0.5:0.95. Among them, EMAF-Net’s mAP@0.5 reaches 45.46%, which is much higher than 42.97% of YOLOv7 and 44.70% of Deformable DETR. Compared with the better-performing YOLOv11 at 45.32%, it has achieved a 0.14% improvement, further consolidating its generalization ability. Additionally, in the more complex scenarios with stricter IoU indicators, mAP@0.5:0.95, EMAF-Net surpasses the other three models with a precision of 27.01, verifying its good adaptability to complex environments. In contrast, YOLOv7, as a classic object detection model, achieves only 42.97% on mAP@0.5, reflecting its still-limited adaptability to complex scenes. Deformable DETR’s overall accuracy is not significantly better than other models, with mAP@0.5:0.95 being only 23.90%, indicating the model’s inadaptability to fine-grained detection requirements. YOLOv11 performs well in multi-scale feature extraction optimization, with mAP@0.5 reaching 45.32%. However, EMAF-Net, with its improved feature fusion strategy and robust design, has better generalization ability, achieving higher precision in detection performance while also having the advantage of a lightweight design.

Overall, EMAF-Net’s performance on the BDD100K dataset not only leads other models in all indicators but also demonstrates its excellent adaptability to complex environments and diverse objects, further verifying the generalization ability of EMAF-Net and the feasibility of model improvement strategies on public datasets and their applicability to real-world scene tasks.

**Figure 13:**
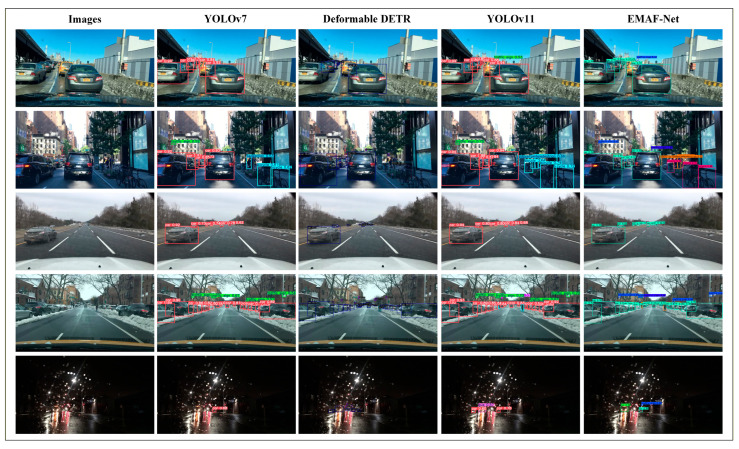
Visualization of object detection results on the BDD100K dataset.

To more intuitively present the comparison results of all models on the public dataset BDD100K, Figure 13 shows the visual detection results of four models in various driving scenarios. The BDD100K dataset includes driving scenes of urban streets and rural highways, and is an important benchmark for evaluating the generalization performance and object detection capabilities of the models. In the detection task, these scenarios pose strict challenges to the models in recognizing distant objects, small objects, occlusions, and dynamic objects.

From the results in Figure 13, it can be seen that different models have their own advantages in object detection in complex scenarios, but EMAF-Net performs the most outstandingly. YOLOv7 shows a better bounding box selection effect in some object detections, such as the recognition of car classes in daytime scenes, which is relatively accurate. However, for small objects like the pedestrian at a long distance in the fourth picture, and the traffic sign, bus, and car in the rainy and dark scene of the fifth picture, they were not accurately recognized, and the confidence levels were generally low. Deformable DETR had generally low confidence levels: a very obvious car in the first picture was not detected, and the traffic sign in the second picture was not recognized either. The confidence level of the car was the highest at only 0.871 in the EMAF-Net. Especially in low-light conditions, the phenomenon of missed detection and false detection was particularly obvious. In the second and fifth pictures, a large number of airplanes appeared as misidentified phenomena by the model, showing obvious limitations. Compared with the previous two models, YOLOv11 significantly improved the detection accuracy for common categories but still had difficulty in capturing distant objects or small objects in complex backgrounds. For example, the traffic light in the second picture was not recognized, the object box of the car at a long distance in the third picture was one less than EMAF-Net, and the traffic sign in the fifth picture was misidentified as a car and was not recognized by YOLOv7.

Compared with the above models, EMAF-Net demonstrated excellent detection performance under different lighting and scene conditions, especially in the detection of small objects, distant objects, and complex category scenes. In the first picture, it could accurately detect multiple object categories, such as traffic signs and cars, and the confidence level was higher than the other models. Especially for traffic signs, its confidence level reached 0.61, significantly surpassing 0.53 of YOLOv11, while the other two models did not recognize it. The confidence level of bicycle detection in the second picture reached 0.91, far exceeding the other models. In addition, the model accurately recognized traffic lights that the other three models did not recognize. Moreover, the traffic sign had a confidence level that was only 0.06 lower than that of YOLOv11, 0.17 higher than that of YOLOv7, and Deformable DETR failed to recognize it. In the fifth picture, it could be clearly seen that in the night and rainy scene, the detection ability of EMAF-Net was particularly outstanding, not only recognizing more categories than other models but also having significantly higher confidence levels.

### 4.5. Ablation Experiments

#### 4.5.1. Ablation Experiment on the Impact of Different Thresholds on Annotation Quality

In order to verify the impact of different IoU thresholds on the quality of data annotation and its correlation with model performance (mAP@0.5:0.95, FNR), this experiment randomly selected 500 images from the annotated dataset as the pre-experiment subset, which included all the categories and scene types required for the experiment. After manual annotation was completed, the annotation files in XML format were exported, and each bounding box annotation was read in batches and its IoU was calculated to generate two sets of data. One set included images with IoU ≥ 0.5, and the other set included images with IoU ≥ 0.75. Then, YOLOv11 was trained on both sets of annotated data separately, ensuring that all hyperparameters (learning rate (lr), batch size (bs), etc.) had the same values. Finally, based on the two indicators of mAP@0.5:0.95 and FNR, the performance of the model under different IoU thresholds was comprehensively evaluated, as shown in Table 4. mAP@0.5:0.95 reflects the average precision of the model, and FNR reflects the proportion of true objects that the model failed to detect out of the total number of objects, and its calculation formula is as follows:

(11) $$FNR=\frac{FN}{FN+TP}$$

FN (False Negative) represents the number of missed detections, which is the quantity of actual objects that the model failed to identify. TP (True Positive) represents the number of correctly identified objects.

**Figure 14:**
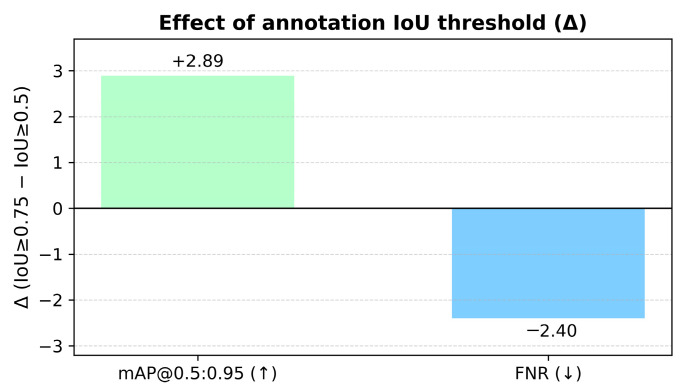
Effect of annotation IoU threshold on performance.

According to the above table, it can be seen that when the IoU threshold is increased, the mAP@0.5:0.95 improves by 2.89%, which indicates that the model can effectively capture the object features under strict bounding box annotation standards and improve the detection accuracy. Moreover, the FNR decreased by 2.4%, which means that the strict annotation standards (IoU ≥ 0.75) force the annotators to closely adhere to the object boundaries, reducing the deviation between the annotated boxes and the real objects, making it easier for the model to learn precise boundary features during training. This implies that the risk of collision caused by the omission of dynamic obstacles (such as people, animals) during the navigation environment perception process of agricultural machinery can be significantly reduced. This discovery provides a direct basis for the optimization of the perception system of safety-sensitive agricultural machinery. By strengthening the quality control of annotations, the reliability of the model in complex scenarios can be effectively improved, and the probability of safety accidents caused by omission can be reduced. Figure 14 shows the impact of different annotation IoU thresholds on performance, where Δ represents the change in IoU ≥ 0.75 relative to IoU ≥ 0.5 ($\Delta =\left\{\mathrm{IoU}\geq 0.75\right\}-\left\{\mathrm{IoU}\geq 0.5\right\}$). The larger the mAP, the better, and the smaller the FNR, the better. Therefore, −Δ represents the reduction in the omission rate.

In addition, this study quantitatively evaluated the impact of annotation strictness on key indicators such as detection performance and false negative rate in rural road scenarios. Compared to the default IoU = 0.5 annotation consistency requirement of general datasets, the IoU ≥ 0.75 annotation standard proposed and verified in this study is more suitable for the irregularity of objects (such as agricultural machinery, farm tools, and free-range animals) and edge blurriness (such as dust from sandy roads and low-light conditions) in agricultural scenarios. It provides reusable annotation norms for the subsequent construction of rural road datasets. Moreover, the experimental results have extension value for our subsequent work on object tracking tasks. High IoU annotated data can improve the positioning accuracy of single-frame detection, enhance the stability of cross-frame object ID association, and thereby improve the trajectory prediction and obstacle avoidance decision-making ability of agricultural machinery during continuous operations. This discovery provides a new optimization direction for the algorithm design of the detection-tracking joint task in agricultural scenarios, that is, through strict quality control in the annotation stage, to reduce the sensitivity of the tracking task to complex environments (such as occlusion, object deformation).

#### 4.5.2. EMHA Module Ablation Experiment

EMAF-Net leaves the original detection framework unchanged and replaces the CSPDarknet used for feature extraction from P5 to P6 with the EMHA module. The EMHA module consists of two main parts, namely EfficientNet-B1 and the MHSA module. The core motivation for this replacement is also the most significant highlight of our research in this paper: the convolutional structure is good at representing local details, but in rural road scenarios for agricultural machinery autonomous driving, there are often problems such as dense small objects, occlusion, and strong interference from background textures, and relying solely on local receptive fields is prone to causing missed detections and category confusion, while the self-attention mechanism can establish long-range dependencies and complement the global semantic modeling ability, and EfficientNet-B1 has better parameter efficiency and inference speed, which is suitable for edge-end deployment requirements. To verify the interaction between the two modules, EfficientNet-B1 and MHSA, ablation experiments were conducted, and the results are presented in Table 5.

Baseline 1: Use EMAF-Net with CSPDarknet as the backbone, without including the EMHA module, as baseline 1.

Model 1-1: In baseline 1, use the EfficientNet-B1 block as the backbone, with the rest of the structure remaining unchanged.

Model 1-2: In baseline 1, add the MHSA block, with the rest of the structure remaining unchanged.

Model 1-3: In baseline 1, add the EMHA module, replace the backbone and add the multi-head self-attention mechanism, with the rest of the structure remaining unchanged.

**Figure 15:**
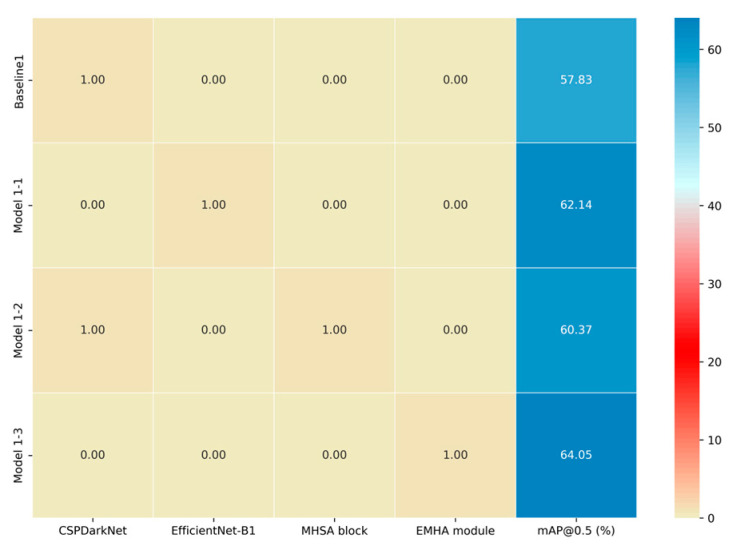
Thermal map of each module’s ablation experiment.

Figure 15 presents a heatmap showing the activation status of each module in the ablation model, and simultaneously provides the corresponding mAP@0.5 values. By referring to Table 5, it shows that based on Baseline 1, replacing the backbone with EfficientNet-B1 (Model 1-1) can increase mAP@0.5 by 4.31%; while introducing only the MHSA block (Model 1-2) results in an mAP@0.5 of 60.37%, which is 1.77% lower than Model 1-1 and 2.54% higher than Baseline 1, indicating that relying solely on attention-enhanced global modeling is not sufficient to fully meet the feature expression requirements in complex textures of rural roads and dense scenes with small objects. Further, when EfficientNet-B1 and MHSA are introduced in the form of EMHA module as Model 1-3, mAP@0.5 reaches 64.05%, which is 6.22% higher than Baseline 1 and 1.91% higher than Model 1-1, indicating that the parameter efficiency and stable local representation ability provided by the efficient convolution backbone, together with the long-range dependency modeling of self-attention, are complementary in this task, and can effectively reduce the false detection and confusion caused by background texture interference. In conclusion, the EMAF-Net integrated with the EMHA module achieves the best detection performance under this set of ablation settings.

#### 4.5.3. EfficientNet Feature Selection Ablation Experiment

To verify the effectiveness of our strategy for selecting the internal feature layers in EfficientNet B1, a set of ablation experiments was set up. In this paper, the seven groups of MBConv stages in EfficientNet B1 are sequentially numbered as stage1–stage7, and three-scale features are selected as the input for the subsequent neck.

Baseline2: The baseline model is EMAF-Net, which uses the EfficientNet-B1 backbone network and does not incorporate the MHSA attention mechanism. The three-scale features use the original stage outputs corresponding to the downsampling ratios of 8, 16, and 32.

Model 2-1: The three-scale feature extraction results are selected as the outputs of the first 1 × 1 convolution kernels in stages 4 and 6, and the end output of the backbone network.

Model 2-2: The three-scale feature extraction results are selected as the outputs of the first 1 × 1 convolution kernels in stages 5 and 7, and the end output of the backbone network.

**Figure 16:**
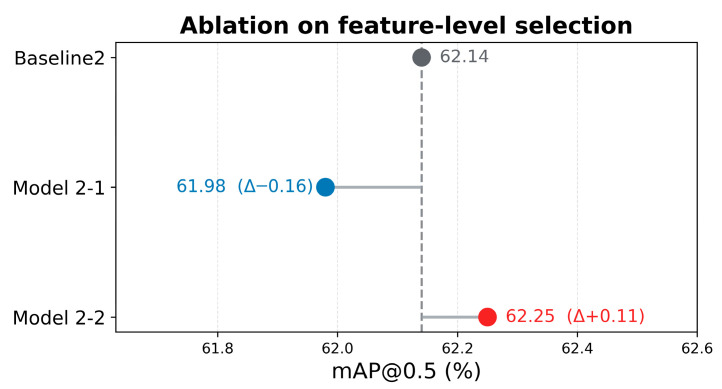
Feature selection strategy.

As shown in Figure 16, in the absence of MHSA, the mAP@0.5 of Baseline2 was 62.14%. The mAP@0.5 of Model 2-1 dropped to 61.98%, indicating that this hierarchical combination is not friendly to multi-scale representation in this task. However, the mAP@0.5 of Model 2-2 increased to 62.25%, which was an improvement of 0.11% compared to Baseline2. This suggests that the output of the dimension expansion layer can provide more discriminative feature representations at a deeper level, thereby achieving stable performance improvement.

## 5. Discussion

This study focuses on the perception scenarios of rural roads in the context of autonomous driving for agricultural machinery. Compared to urban roads, rural road images typically have stronger unstructured features and higher uncertainty: background textures present significant interference (vegetation, dust, gravel and debris), the lighting and weather conditions are highly variable (strong light/shadow, rain and fog, night low light), object occlusions are more frequent, and the proportion of distant small objects and objects with varying scales is higher. These factors collectively lead to an increase in the appearance variation of similar objects and a weakening of the discriminative cues between different categories, making traditional detectors based on local convolution more prone to missed detections and category confusion, especially in scenarios with dense small objects, occlusion, and complex backgrounds. Under a unified input resolution (640 × 640) and training settings, EMAF-Net achieved 64.05% mAP@0.5 on the self-built rural road dataset, outperforming YOLOv7 (55.52%), YOLOv11 (61.94%) and Deformable DETR (50.90%). More importantly, this improvement in accuracy was not achieved by increasing the model size: EMAF-Net has only 18.3 M parameters and 38.5GFLOPs and is significantly lower in complexity than YOLOv11 and YOLOv7. The 184.62 FPS obtained after exporting to ONNX on RTX 4090 further indicates that this structure has a high throughput potential; however, it should be emphasized that the actual deployment speed on edge devices is still closely related to the inference accuracy mode, preprocessing method, hardware backend (TensorRT/NPU, etc.) and navigation system. A more application-oriented latency assessment under a unified protocol still needs to be provided in the future.

From the model perspective, the performance improvement of EMAF-Net mainly stems from the EMHA design of the backbone network. Its value lies not in introducing attention but in the synergy between attention and the high-parameter-efficient convolutional backbone: the MBConv of EfficientNet-B1 can still provide stable local texture and edge representation with fewer parameters and computational costs, while MHSA can establish long-range dependencies across spatial locations, alleviating the feature fragmentation problem that is prone to occur when relying solely on local convolution for dense small objects and occluded objects. In other words, EMHA does not replace convolution with attention but uses the local representation capability of convolution as the foundation, and then uses attention to enhance global semantic alignment and inter-regional association modeling, thereby better adapting to the feature distribution with strong interference in rural road backgrounds and large variations in object scales.

From the perspective of error diagnosis, the confusion matrix in Figure 9 shows that the remaining errors mainly concentrate among categories with similar appearances. For instance, the confusion ratio between motorcycle and tricycle is 0.07. Such confusion is consistent with the imaging characteristics of rural roads: the long-distance frontal view compresses the differences in shapes, and the presence of people or objects leads to the occlusion of key structures, thereby weakening the fine-grained discrimination cues. Therefore, the global modeling of EMHA helps alleviate the missed detections caused by complex backgrounds and occlusions, but there is still room for improvement for categories with small fine-grained differences and highly similar appearances. In the future, more fine-grained supervision signals or objective enhancement strategies can be introduced.

The scale distribution in rural road scenarios spans a wider range: it includes both nearby large vehicles/agricultural machinery and distant small objects (such as pedestrians, traffic signs, and street lights). If the model relies solely on single-scale or unidirectional pyramid fusion, it is prone to issues such as insufficient detail for small objects or ineffective back-propagation of high-level semantics to low-level localization. In this paper, an Improved ASPP and bidirectional FPN are introduced in the neck, with the following significance: expanding the effective receptive field and supplementing multi-scale contextual information through multi-branch dilated convolution and pyramid pooling, and then enhancing the alignment between semantics and details through bidirectional cross-layer fusion, thereby improving scale robustness and stability in small object detection. Combined with the complexity comparison in Table 2, EMAF-Net achieves higher mAP while maintaining significantly lower GFLOPs than YOLOv11, which can be regarded as a more computationally efficient multi-scale enhancement scheme.

From a data perspective, the IoU ≥ 0.75 annotation consistency quality control proposed and verified in this paper is not merely a preference for annotation but a key factor that can significantly affect the stability of training supervision. Since the mAP@0.5:0.95 metric is more sensitive to positioning accuracy, if the annotation boxes are too loose or too tight, it will introduce regression supervision noise and reduce the model’s learning stability for boundaries. In rural road data, a large number of object boundaries are irregular or blurred due to low light, dust, or other factors (such as farm tools, animals, and night objects), and even a small deviation in annotation can lead to significant fluctuations in IoU, thereby amplifying training noise. Therefore, the improvement in mAP@0.5:0.95 brought about by stricter consistency standards and the reduction in missed detections are reasonable and reusable, providing an operational quality control specification for the subsequent construction of rural road datasets and laying a more stable single-frame positioning foundation for future detection-tracking joint tasks.

On the BDD100K dataset, EMAF-Net achieved an mAP@0.5 of 45.46% with an input size of 640 × 640, which was a slight improvement compared to YOLOv11 and outperformed YOLOv7 and Deformable DETR. This result indicates that the structure proposed in this paper is not only overfitting-specifically effective for the self-built rural road object detection dataset but also has certain cross-domain robustness. At the same time, the limited improvement is in line with expectations: BDD100K is more inclined towards urban/general road categories and scene priors, and the advantages of agricultural-related categories are difficult to demonstrate; moreover, YOLOv11 already has strong baseline performance in general detection tasks, resulting in a smaller marginal benefit from structural improvements. To further improve the cross-domain accuracy, domain-adaptive training, style enhancement for weather/lighting conditions, and re-weighting strategies for small objects can be combined to enhance the stability across data distributions.

Although EMAF-Net achieves a relatively optimal balance between accuracy, complexity and speed, it still has several limitations: (1) Fine-grained confusion among similar categories still exists. Subsequent studies could consider introducing hierarchical classification (coarse-to-fine), re-weighted loss for the confusion pairs, or stronger occlusion/viewpoint enhancement to strengthen the fine-grained discrimination features. (2) The reliability in extreme low-light/rainy/foggy conditions may still be limited, especially in the contrast visualization, where some models exhibit a decrease in confidence. Further exploration of low-light enhancement, combined training for rain and fog removal, or the introduction of multi-frame information fusion could be conducted to improve robustness. (3) Long-tail categories and irregular objects (such as farm tools, animals, etc.) have higher learning difficulty under ambiguous boundaries and morphological changes. Future work could adopt class-balanced loss, pseudo-label semi-supervised expansion, or active learning-based data sampling to improve category recognition accuracy. Furthermore, data augmentation strategies based on GANs [29] can be further explored to synthesize diverse training samples, thereby alleviating issues of class imbalance and limited sample size. (4) For the continuous perception requirements of agricultural machinery autonomous driving, subsequent studies could combine detection-tracking joint learning and trajectory prediction to enhance the ability to recover from occlusions and the stability of decision-making for dynamic obstacles. (5) The FPS was verified in this paper for the structure throughput potential on RTX 4090, but end-side deployment still requires systematic evaluation combined with TensorRT/FP16/INT8 quantization and specific hardware (such as Jetson/NPU) and the exploration of block inference (tiling) or multi-scale strategies to balance accuracy and real-time performance by leveraging the advantages of 4K data acquisition.

## 6. Conclusions

Rural road scenes have features such as complex background textures, drastic changes in lighting and weather, large-scale variations of objects, and frequent occlusions, which make general detectors based on conventional convolutional backbones prone to missing detections and confusion in small objects and similar category discrimination. In response to the real-time obstacle perception requirements for agricultural machinery automatic navigation, this paper constructs a 4K rural road object detection dataset covering 13 types of objects, and proposes a lightweight and efficient single-stage detection network, EMAF-Net. This model replaces the CSPDarknet in the YOLOv4-P6 framework with the EMHA module, enabling the complementarity of EfficientNet-B1’s efficient local representation and MHSA’s global dependency modeling. At the neck, Improved ASPP and FPN are used for multi-scale feature fusion, and the CIoU loss is combined to improve the localization quality. Experimental results show that EMAF-Net achieves 64.05% mAP@0.5 and 48.95% mAP@0.5:0.95 on the self-built dataset, with 18.3 M parameters and 38.5GFLOPs. After converting to ONNX format on RTX 4090, the inference speed reaches 184.62 FPS. In addition, to evaluate the feasibility of real-world deployment, inference experiments were conducted on a representative edge-level GPU (NVIDIA 1650Ti), where EMAF-Net achieves an average speed of approximately 27 FPS (39 ms per frame), meeting the real-time perception requirements for agricultural machinery operating in rural road environments. The comprehensive performance is superior to YOLOv7, YOLOv11, and Deformable DETR, demonstrating good accuracy–efficiency balance and engineering deployment potential. On the BDD100K dataset, it also achieves 45.46% mAP@0.5 and 27.01% mAP@0.5:0.95, demonstrating good cross-domain generalization ability. Ablation experiments further verify that the EMHA module collaboration brings a 6.22% mAP@0.5 improvement, and a strict annotation consistency threshold (IoU ≥ 0.75) can significantly improve mAP@0.5:0.95 and reduce the missed detection rate, providing reusable specifications for agricultural scene dataset construction. Future work will introduce detection-tracking multi-task joint learning and trajectory prediction to enhance the continuous perception ability of dynamic obstacles, expand the diversity of regional and seasonal scene data, and study stronger cross-domain adaptability and small-sample transfer strategies, thereby further enhancing the model’s robustness in different rural road conditions.

## Figures and Tables

**Table 1:** Comparison of dataset label contributions.

Dataset	Different Lighting		Different Road Features		Different Weather	Category	Image Resolution
	Daytime	Nighttime	Structured Roads	Unstructured Roads			
BDD100K	√	√	√	×	√	10	1280 × 720
D2-City	√	√	√	×	√	12	1280 × 720, 1920 × 1080
Caltech Pedestrian	√	×	√	×	×	4	640 × 480
CityPersons	√	×	√	×	√	4	2048 × 1024
NightOwls	×	√	√	×	√	4	1024 × 640
**Ours**	**√**	**√**	**√**	**√**	**√**	**13**	**3840 × 2160**

**Table 2:** Quantitative comparison of results for all models on the rural road dataset.

Model	Input Size	#Params (M)	GFLOPs	Epoch	FPS	mAP@0.5 (%)	mAP@0.5:0.95 (%)
YOLOv7	640 × 640	36.9	104.7	300	157.02	55.52	41.39
Deformable DETR	-	40.0	173.0	300	47.30	50.90	30.50
YOLOv11	640 × 640	20.1	68.0	300	132.84	61.94	44.32
**EMAF-Net**	**640 × 640**	**18.3**	**38.5**	**300**	**184.62**	**64.05**	**48.95**

**Table 3:** Quantitative comparison of results for all models on the BDD100K dataset.

Model	Input Size	Epoch	mAP@0.5 (%)	mAP@0.5:0.95 (%)
YOLOv7	640 × 640	150	42.97	24.06
Deformable DETR	-	150	44.70	23.90
YOLOv11	640 × 640	150	45.32	26.88
**EMAF-Net**	**640 × 640**	**150**	**45.46**	**27.01**

**Table 4:** Impact of different IoU thresholds on rural road object detection performance.

Annotation IoU Threshold	mAP@0.5:0.95 (%)	FNR (%)	Params (M)
IoU ≥ 0.5	31.27	18.2	20.1
**IoU ≥ 0.75**	**34.16**	**15.8**	**20.1**

**Table 5:** Results of ablation experiments for the EMHA module.

Model	CSPDarkNet	EfficientNet-B1	MHSA Block	EMHA Module	mAP@0.5 (%)
Baseline1	√				57.83
Model 1-1		√			62.14
Model 1-2	√		√		60.37
**Model 1-3**				**√**	**64.05**

## Data Availability

The raw data supporting the conclusions of this article will be made available by the authors on request.

## Abbreviations

The following abbreviations are used in this manuscript:

MHSA	Multi-head self-attention
FPN	Feature pyramid networks
ASPP	Atrous spatial pyramid pooling
SAA	Spatial adaptive alignment
ELAN	Efficient layer aggregation network
E-ELAN	Extended efficient layer aggregation networks
SNR	Signal-to-noise ratio
FNR	False negative rate
CNN	Convolutional neural network
DVC	Distributed version control
CIoU	Complete intersection over union
MBConv	Mobile Inverted Bottleneck Convolution
SE	Squeeze and excitation
MSE	Mean square error
GFLOPs	Giga floating-point operations per second
lr	Learning rate
bs	Batch size
FN	False negative
TP	True positive

## References

[1] Bai Y., Zhang B., Xu N., Zhou J., Shi J., Diao Z. (2023). Vision-based navigation and guidance for agricultural autonomous vehicles and robots: A review. Comput. Electron. Agric..

[2] Barba-Guaman L., Naranjo J.E., Ortiz A., Gonzalez J.G.P. (2021). Object detection in rural roads through SSD and YOLO framework. Proceedings of the World Conference on Information Systems and Technologies, Azores, Portugal, 30 March–1 April 2021.

[3] Li S., Xu H., Ji Y., Cao R., Zhang M., Li H. (2019). Development of a following agricultural machinery automatic navigation system. Comput. Electron. Agric..

[4] Gao C., Zhao F., Zhang Y., Wan M. (2024). Research on multitask model of object detection and road segmentation in unstructured road scenes. Meas. Sci. Technol..

[5] Murthy J.S., Siddesh G.M., Lai W.-C., Parameshachari B.D., Patil S.N., Hemalatha K.L. (2022). ObjectDetect: A Real-Time Object Detection Framework for Advanced Driver Assistant Systems Using YOLOv5. Wirel. Commun. Mob. Comput..

[6] Yao Z.-X., Wang H., Meng Z.-J., Yang L.-L., Zhang T.-H. (2025). Multi-scale feature alignment network for 19-class semantic segmentation in agricultural environments. Artif. Intell. Agric..

[7] Geetha A.S. (2025). YOLOv4: A Breakthrough in Real-Time Object Detection. arXiv.

[8] Yu F., Chen H., Wang X., Xian W., Chen Y., Liu F., Madhavan V., Darrell T. Bdd100k: A diverse driving dataset for heterogeneous multitask learning. Proceedings of the IEEE/CVF Conference on Computer Vision and Pattern Recognition.

[9] Zou Z., Chen K., Shi Z., Guo Y., Ye J. (2023). Object detection in 20 years: A survey. Proc. IEEE.

[10] Redmon J., Divvala S., Girshick R., Farhadi A. (2016). You only look once: Unified, real-time object detection. Proceedings of the IEEE Conference on Computer Vision and Pattern Recognition, Las Vegas, NV, USA, 27–30 June 2016.

[11] Lin T.Y., Dollár P., Girshick R., He K., Hariharan B., Belongie S. (2017). Feature pyramid networks for object detection. Proceedings of the IEEE Conference on Computer Vision and Pattern Recognition, Honolulu, HI, USA, 21–26 July 2017.

[12] Lin T., Goyal P., Girshick R., He K., Dollár P. (2017). Focal loss for dense object detection. Proceedings of the IEEE International Conference on Computer Vision, Venice, Italy, 22–29 October 2017.

[13] Law H., Deng J. (2018). Cornernet: Detecting objects as paired keypoints. Proceedings of the European Conference on Computer Vision (ECCV), Munich, Germany, 8–14 September 2018.

[14] Chen J., Bai T. (2020). SAANet: Spatial adaptive alignment network for object detection in automatic driving. Image Vis. Comput..

[15] Cai Y., Luan T., Gao H., Wang H., Chen L., Li Y., Sotelo M.A., Li Z. (2021). YOLOv4-5D: An effective and efficient object detector for autonomous driving. IEEE Trans. Instrum. Meas..

[16] Wang C.Y., Bochkovskiy A., Liao H.Y.M. (2023). YOLOv7: Trainable bag-of-freebies sets new state-of-the-art for real-time object detectors. Proceedings of the IEEE/CVF Conference on Computer Vision and Pattern Recognition, Vancouver, Canada, 18 June–22 June 2023.

[17] Carion N., Massa F., Synnaeve G., Usunier N., Kirillov A., Zagoruyko S. (2020). End-to-end object detection with transformers. Proceedings of the European Conference on Computer Vision, Glasgow, UK, 23–28 August 2020.

[18] Meng D., Chen X., Fan Z., Zeng G., Li H., Yuan Y., Sun L., Wang J. (2021). Conditional detr for fast training convergence. Proceedings of the IEEE/CVF International Conference on Computer Vision, Montreal, QC, Canada, 11–17 October 2021.

[19] Zhao Y., Lv W., Xu S., Wei J., Wang G., Dang Q., Liu Y., Chen J. (2024). Detrs beat yolos on real-time object detection. Proceedings of the IEEE/CVF Conference on Computer Vision and Pattern Recognition, Seattle, WA, USA, 17–21 June 2024.

[20] Zhu X., Su W., Lu L., Li B., Wang X., Dai J. (2020). Deformable detr: Deformable transformers for end-to-end object detection. arXiv.

[21] Dai Z., Liu H., Le Q.V., Tan M. (2021). Coatnet: Marrying convolution and attention for all data sizes. Adv. Neural Inf. Process. Syst..

[22] Li Y., Yuan G., Wen Y., Hu J., Evangelidis G., Tulyakov S., Wang Y., Ren J. (2022). Efficientformer: Vision transformers at mobilenet speed. Adv. Neural Inf. Process. Syst..

[23] Yao Z., Zhao C., Zhang T. (2024). Agricultural machinery automatic navigation technology. iScience.

[24] Buslaev A., Iglovikov V.I., Khvedchenya E., Parinov A., Druzhinin M., Kalinin A.A. (2020). Albumentations: Fast and flexible image augmentations. Information.

[25] Maharana K., Mondal S., Nemade B. (2022). A review: Data pre-processing and data augmentation techniques. Glob. Transit. Proc..

[26] Zhang Z., Lu X., Cao G., Yang Y., Jiao L., Liu F. (2021). ViT-YOLO: Transformer-based YOLO for object detection. Proceedings of the IEEE/CVF International Conference on Computer Vision, Montreal, QC, Canada, 11–17 October 2021.

[27] Tan M., Le Q. (2019). Efficientnet: Rethinking model scaling for convolutional neural networks. Proceedings of the 36th International Conference on Machine Learning, Long Beach, CA, USA, 10–15 June 2019.

[28] Khanam R., Hussain M. (2024). Yolov11: An overview of the key architectural enhancements. arXiv.

[29] Ranjan P., Nandal A., Agarwal S., Kumar R. (2026). A Dive into Generative Adversarial Networks in the World of Hyperspectral Imaging: A Survey of the State of the Art. Remote Sens..

